# Calcium channel CNGC19 mediates basal defense signaling to regulate colonization by *Piriformospora indica* in Arabidopsis roots

**DOI:** 10.1093/jxb/eraa028

**Published:** 2020-01-20

**Authors:** Abhimanyu Jogawat, Mukesh Kumar Meena, Anish Kundu, Mahendra Varma, Jyothilakshmi Vadassery

**Affiliations:** 1 National Institute of Plant Genome Research, Aruna Asaf Ali Marg, New Delhi, India; 2 University of Birmingham, UK

**Keywords:** *Arabidopsis thaliana*, callose, cell-wall extract, cellotriose, CNGC19, indole glucosinolates, phytohormones, *Piriformospora indica*, *Serendipita indica*

## Abstract

The activation of calcium signaling is a crucial event for perceiving environmental stress. Colonization by *Piriformospora indica*, a growth-promoting root endosymbiont, activates cytosolic Ca^2+^ in Arabidopsis roots. In this study, we examined the role and functional relevance of calcium channels responsible for Ca^2+^ fluxes. Expression profiling revealed that *CYCLIC NUCLEOTIDE GATED CHANNEL 19* (*CNGC19*) is an early-activated gene, induced by unidentified components in *P. indica* cell-wall extract. Functional analysis showed that loss-of-function of CNGC19 resulted in growth inhibition by *P.indica*, due to increased colonization and loss of controlled fungal growth. The *cngc19* mutant showed reduced elevation of cytosolic Ca^2+^ in response to *P. indica* cell-wall extract in comparison to the wild-type. Microbe-associated molecular pattern-triggered immunity was compromised in the *cngc19* lines, as evidenced by unaltered callose deposition, reduced *cis*-(+)-12-oxo-phytodienoic acid, jasmonate, and jasmonoyl isoleucine levels, and down-regulation of jasmonate and other defense-related genes, which contributed to a shift towards a pathogenic response. Loss-of-function of CNGC19 resulted in an inability to modulate indole glucosinolate content during *P. indica* colonization. CNGC19-mediated basal immunity was dependent on the AtPep receptor, PEPR. CNGC19 was also crucial for *P. indica*-mediated suppression of AtPep-induced immunity. Our results thus demonstrate that Arabidopsis CNGC19 is an important Ca^2+^ channel that maintains a robust innate immunity and is crucial for growth-promotion signaling upon colonization by *P. indica*.

## Teaser

The CNGC19 calcium channel plays an important role in regulating the colonization of Arabidopsis roots by *Piriformospora indica* via modulation of systemic and defense-related pathways of MAMP-triggered immunity.

## Introduction

*Piriformospora indica* (syn. *Serendipita indica*) is a cultivable, root-colonizing endophytic fungus belonging to Sebacinales (Basidiomycota) (Verma *et al.*, 1998; Weiß *et al.*, 2016). It colonizes many plant species including Arabidopsis and promotes their growth (Varma *et al.*, 1999; Peškan‐Berghöfer *et al.*, 2004; Vadassery *et al.*, 2009), enhances nutrient uptake (Yadav *et al.*, 2010; Rani *et al.*, 2016; Bakshi *et al.*, 2017; Prasad *et al.*, 2018), and imparts tolerance to abiotic and biotic stresses to a wide range of its hosts (Waller *et al.*, 2005; Baltruschat *et al.*, 2008; Jogawat et al., 2013, 2016; Sun *et al.*, 2014). It colonizes the root epidermal and cortex cells without penetrating the central cylinder, and displays a biphasic colonization strategy (Deshmukh *et al.*, 2006; Zuccaro *et al.*, 2011). However, the establishment of a beneficial plant–microbe interaction is not always harmonious, and rejection of the invading symbiont or control of its colonization can occur due to active plant defense (Vadassery and Oelmüller, 2009). Basal plant defense relies on the recognition of conserved microbial structures called microbe-associated molecular patterns (MAMPs), and is termed MAMP-triggered immunity (MTI) (Millet *et al.*, 2010). In soil, plant roots perceive MAMPs using specific pattern-recognition receptor (PRR) proteins (Choi and Klessig, 2016). Recognition of MAMPs triggers downstream early plant-defense responses such as elevation of cytosolic calcium (Ca^2+^_cyt_) and a burst of reactive oxygen species (ROS), which further activates mitogen-activated protein (MAP) kinase and various phytohormone pathways that stimulate defense-related pathways (Harper and Harmon, 2005; Ranf *et al.*, 2011; Steinhorst and Kudla, 2013). Similar to plant pathogens, mutualists such as *P. indica* are also confronted with an effective innate immune system in roots, and the colonization success depends on the evolution of strategies for immunosuppression (Van Wees *et al.*, 2008; Jacobs *et al.*, 2011). During colonization by *Serendipita indica* on Arabidopsis, eATP, which acts as a damage-associated molecular pattern (DAMP) accumulates in the apoplast. *Serendipita indica* secrets *Si*E5′NT, an enzymatically active nucleotidase capable of hydrolysing eATP, in the apoplast and thus suppresses immunity (Nizam *et al.*, 2019). During the early stages of mycorrhiza formation and *P. indica* colonization, H_2_O_2_ is produced and its production declines when a mutualistic interaction is established (Fester and Hause, 2005; Matsuo *et al.*, 2015). *Piriformospora indica* also actively represseses ROS accumulation by activating ROS-scavenging genes (Matsuo *et al.*, 2015). To achieve a harmonious interaction with plants, *P. indica* also regulates biosynthesis and signaling of several phytohormones such as jasmonic acid (JA), gibberallins (GA), and ethylene (Camehl *et al.*, 2010; Sun *et al.*, 2014; Vahabi *et al.*, 2015; Pan *et al.*, 2017; Xu *et al.*, 2018). *Piriformospora indica* association also alters callose deposition and defense-related metabolites, such as phytoalexins and glucosinolates (GS) (Jacobs *et al.*, 2011; Lahrmann *et al.*, 2015). Indole glucosinolates (iGS) are an important part of MTI in plants (Clay *et al.*, 2009; Böhm *et al.*, 2014) and they are found to be essential in balancing the beneficial interaction between *P. indica* and Arabidopsis (Nongbri *et al.*, 2012; Lahrmann *et al.*, 2015). In addition, *P. indica* also suppresses innate immunity upon encountering the *flagellin 22* elicitor from bacteria (Jacobs *et al.*, 2011). An active plant immunity and its suppression is thus critical for controlled *P. indica* colonization. The early-activated plant defense genes that are responsible for regulating the entry of the symbiont and its subsequent colonization are unknown.

Ca^2+^ is a universal second messenger, activated very early in signaling cascades upon recognition of both pathogens and symbionts. Rhizobacteria-mediated nodulation and mycorrhiza formation are associated with oscillations in nuclear Ca^2+^ in host plants. These oscillations upon perception of rhizobia and mycorrhiza activate induction of the common genes that are important for the establishment of the symbioses (Oldroyd, 2013). Colonization by *P. indica* in Arabidopsis is independent of these common arbuscular mycorrhizal symbiotic genes (Banhara *et al.*, 2015). However, elevation of Ca^2+^ is common with other symbiotic interactions, as *P. indica* cell-wall extract (PiCWE) elevates root Ca^2+^_cyt_ and is crucial for growth promotion in Arabidopsis (Vadassery *et al.*, 2009). Using the elevation of Ca^2+^ as a marker, Johnson *et al.* (2018) identified cellotriose (CT) as the major elicitor in crude PiCWE. It was further confirmed that CT targets a poly(A)-specific ribonuclease in order to modulate plant responses such as elevation of Ca^2+^_cyt_, generation of ROS, expression of defense-related genes, phytohormonal signaling, and growth promotion. Elevation of Ca^2+^_cyt_ requires entry of Ca^2+^ either across the plasma membrane or from intracellular compartments. In Arabidopsis, ligand-gated channels such as cyclic nucleotide gated channels (CNGCs), glutamate receptor-like channels (GLRs), stretch-activated Ca^2+^ channels (OSCAs), and the MID1-complementing activity (MCA) families are the four main plasma membrane Ca^2+^-permeable channels, whilst the slow vacuolar two-pore channel 1 (TPC1) is the key vacuolar channel (Dodd *et al.*, 2010). The Arabidopsis genome encodes 20 members of the CNGC family, with roles in plant development and a functions related to biotic and biotic stresses (Meena and Vadassery, 2015; DeFalco *et al.*, 2016). *CNGC2*, *CNGC4*, *CNGC11*, and *CNGC12* have been reported to play crucial roles in defense against bacterial and fungal pathogens (Yoshioka *et al.*, 2001; Ahn, 2007), and we have recently identified a role of the CNGC19 Ca^2+^ channel in herbivory-induced Ca^2+^ flux and plant defense against *Spodoptera litura* (Meena *et al.*, 2019).

CNGC15 has been identified as critical nuclear channel that generates oscillatory Ca^2+^ signals during arbuscular mycorrhizal symbiosis with *Medicago trancatula* roots (Charpentier *et al.*, 2016). In *Lotus japonicus*, a mutation in the *AtCNGC19* homolog *BRUSH* is reported to result in impaired infection by nitrogen-fixing rhizobia due to a leaky channel (Chiasson *et al.*, 2017). The identity of the channel involved in the elevation of Ca^2+^_cyt_ that is induced by *P. indica* is not yet known. The *P. indica* elicitor CT induces expression of GLR Ca^2+^ channels in Arabidopsis roots; however, (Johnson *et al.*, 2018) found no functional roles for GLR3.3, GLR2.4, GLR2.5, and TPC1 in this response. Expression levels of CNGCs are altered upon PiCWE treatment in plant roots and these are the only other type of Ca^2+^ channel known to be involved in the interaction (Vadassery *et al.*, 2009). We therefore hypothesized that CNGCs might be involved in the generation of elevated Ca^2+^ in Arabidopsis roots and in the downstream signaling in response to *P. indica* mutualism. Our results point to a role of Arabidopsis CNGC19 as an important gatekeeper to regulate *P. indica* colonization.

## Materials and methods

### Plant and fungal material and conditions

*Piriformospora indica* (Verma *et al.*, 1998) was grown and maintained on Kaefer’s medium at 28±2 °C at 110 rpm (Varma *et al.*, 1999; Hill and Kafer, 2001). For *P. indica* co-cultivation we used *Arabidopsis thaliana* wild-type Columbia (Col-0), the T-DNA mutant lines of *AtCNGC19* (At3g17690) SALK_129200C (*cngc19-2*) and SALK_027306 (*cngc19-1*), which were provided by TAIR (Alonso *et al.*, 2003), and the *pepr1 pepr2* double-mutant line provided by Prof. Gerald Berkowitz (University of Connecticut, USA). Adult plants were grown at 22 °C with a 10/14 h light/dark photoperiod and a light intensity of 150 µmol m^−2^ s^−1^ in a growth room (Percival Scientific). For Ca^2+^ measurements, we used transgenic Col-0 expressing cytosolic apoaequorin, (referred to as WT*::aeq*; Knight *et al.*, 1997), and the *cngc19* and *pepr1 pepr2* mutants transformed with the pMAQ2 vector (referred to as *cngc19::aeq* and *pepr1 pepr2::aeq*, respectively. The T_2_ generation was used.

### Plant and fungal interactions in soil and co-cultivation media

For soil experiments, seeds were sown in pots containing soilrite, Irish peat moss, and exfoliated vermiculite (1:1:1, w:w:w) and kept for 2 d at 4 °C in the dark for stratification. The soil was pre-mixed with 1% *P. indica* mycelia (w/w), and plants were grown for 6 weeks after stratification. Control plants had no *P. indica* mycelia in the soil. The plants were grown at 22 °C with a 10/14 h light/dark photoperiod and a light intensity of 150 µmol m^−2^ s^−1^ in the growth room. Samples were harvested at 42 d post-inoculation (dpi).

For plate experiments, seeds were surface-sterilized, stratified under the conditions described above, and placed on half-strength MS plates supplemented with 1% sucrose and 0.8% agar, and germinated for 7 d. The seedlings were grown at 22 °C with a 10/14 h light/dark photoperiod and a light intensity of 150 µmol m^−2^ s^−1^ in the growth room. They were then transferred to 1× PNM medium for co-cultivation with *P. indica* discs for 14 d (Johnson *et al.*, 2011) under similar conditions. Samples were harvested at 2, 7, and 14 dpi.

### Preparation of *P. indica* cell-wall extract and application on roots

*Piriformospora indica* cell-wall extract (PiCWE) was prepared as described by Vadassery *et al.*, 2009. In brief, the mycelia from 14-d-old liquid cultures were homogenized, filtered using nylon membranes, and washed three times with water, twice with chloroform/methanol (1:1), and finally twice with acetone. The mycelial cell wall material obtained was dried at room temperature, suspended in water, and autoclaved for 30 min at 121 °C. It was then filter-sterilized using a 0.22-μM filter, and 50 µl of the resulting extract was used per seedling root for experiments. The active elicitor of PiCWE was recently identified as cellotriose (CT; Johnson *et al.*, 2018). CT (Sigma, C1167) and cellobiose (Sigma, C7252) were used for some experiments.

### Plant treatments and gene expression analysis

Seedlings at 10 d old were treated with 100 μl PiCWE or 10 µM CT by adding to the MS media and were harvested at 0, 15, 30, 45, and 60 min. For the co-cultivation experiment, the seedlings were harvested at 2, 7, and 14 dpi. Each sample consisted of six seedlings and was ground to a fine powder in liquid N_2_, and total RNA was isolated using TRIzol Reagent (Invitrogen) according to the manufacturer’s protocol. Four replicate samples were used. An additional DNAse (Turbo DNAse, Ambion) treatment was included to eliminate any contaminating DNA. cDNA synthesis was performed using a High Capacity cDNA kit (Applied Biosystems). Gene-specific primers were designed using the NCBI primer design tool (http://www.ncbi.nlm.nih.gov/tools/primer-blast) and are listed in Supplementary Table S1 at *JXB* online. qRT-PCR was performed in optical 96-well plates on a CFX96 Real-Time PCR Detection System (Bio-Rad) using iTaq universal SYBR green Mix (Bio-Rad). *AtActin2* (At3g18780) was used as the endogenous control for normalization of transcripts. The fold-induction values of the target genes were calculated using the ΔΔ*C*_T_ method (Livak and Schmittgen, 2001) and were expressed relative to the mRNA level of the genes in the control seedlings, the values of which were set as 1.

### Detection and measurement of *P. indica* colonization

For tracking of colonization, a green fluorescent protein (GFP)-tagged *P. indica* strain was utilized (Hilbert *et al.*, 2012). Roots colonized with tagged *P. indica* were harvested at 2, 7, and 14 dpi, and were cleaned, mounted, and observed using fluorescence microscopy (Nikon 80i). For confocal microscopy, the roots were treated with propidium iodide and observed under a confocal microscope (Leica TCS M5) at an emission wavelength of 505–530 nm with excitation at 470 nm and digital sectioning of 4–5 µm of root thickness. The relative amount of fungal DNA was determined using real time-qPCR utilizing Arabidopsis *Actin2* (At3g18780) and *P. indica Tef1* (Bütehorn *et al.*, 2000). Relative changes in fungal DNA content were calculated using the *C*_T_ values of *PiTef1*, which were normalized by the *C*_T_ values of *AtActin2* using the ΔΔ*C*_T_ equation and setting the *P. indica* DNA content of the control roots as 1 (Vadassery *et al.*, 2008).

### Tissue localization by GUS assays

Transgenics with a fusion of the *CNGC19* promoter and β-glucuronidase (GUS) were constructed as previously described by Meena *et al.*, 2019. Arabidopsis seedlings of *ProCNGC19::GUS*-expressing transgenic plants (T_3_ generation) were co-cultivated with *P. indica*, and were carefully harvested at 2 dpi and 7 dpi. They were vacuum-infiltrated with GUS staining solution and incubated in the dark at 37 °C. Tissues were decolored by treating with 70% ethanol at 65 °C and then observed under a light microscope (Nikon 80i).

### Measurements of elevation of cytoplasmic Ca^2+^

WT*::aeq*, *cngc19-2::aeq*, and *pepr1 pepr2::aeq* seedlings (Meena *et al.*, 2019) were grown on MS medium and roots of 14-d-old seedlings were transferred to a 96-well white plate (ThermoFisher Scientific) containing 5 μM coelenterazine (PJK, Germany) and left in the dark overnight at 21 °C. Bioluminescence counts in the roots were recorded as relative light units (RLU) per second using a microplate luminometer (Luminoscan Ascent, v. 2.6, ThermoFisher Scientific). After a 1-min background reading, PiCWE (50 µl), cellobiose (100 µM), or CT (10 µM) was added and readings were taken for 15 min. Discharge solution (1 M CaCl_2_ and 10% ethanol) was used for calibrations to estimate the aequorin that remained at the end of the experiment (Vadassery *et al.*, 2012; Meena *et al.*, 2019). The luminescence counts obtained were calibrated using the equation presented by Rentel and Knight (2004).

### Glucosinolate analysis

For analysis of glucosinolates (GS), samples of plants were harvested at 2, 14, and 42 dpi. At 2 dpi and 14 dpi, 40 whole seedlings per replicate were harvested, whilst at 42 dpi whole rosettes were harvested. Four replicate samples were used at all time points.. The samples were frozen in liquid N_2_, lyophilized, and ground to a fine powder in TissueLyser II (Qiagen). Total GSs were extracted with 80% methanol solution containing 0.05 mM 4-hydroxybenzylglucosinolate as an internal standard. Extracts were loaded onto DEAE Sephadex A 25 columns and treated with arylsulfatase for desulfation (Sigma-Aldrich). The eluted desulfoglucosinolates were separated using HPLC (Shimadzu CLASS-VP V6.14) on a reversed-phase C-18 column (250×4.6 mm with 0.5 μm internal diameter) with a water–acetonitrile gradient as follows: 0–1.5% acetonitrile from 0–1 min, 1.5–5% acetonitrile from 1–6 min, 5–7% acetonitrile from 6–8 min, 7–21% acetonitrile from 8–18 min, 21–29% acetonitrile from 18–23 min, 29–100% acetonitrile from 23–24 min, 100–1.5% acetonitrile from 24–28 min. This was followed by a washing cycle with a flow of 1 ml min^−1^ (Vadassery *et al.*, 2012). Detection was performed using a photodiode array detector and peaks were integrated at 229 nm. The following response factors were used for quantification of individual glucosinolates: aliphatic glucosinolates, 2.0; indole glucosinolates, 0.5; and 2-phenylethyl glucosinolate, 2.0 (Burow *et al.*, 2006).

### Estimation of phytohormones

Phytohormones were quantified as described previously (Vadassery *et al.*, 2012; Meena *et al.*, 2019). Seedlings were sampled at 2 dpi and 14 dpi. The samples were harvested, frozen immediately in liquid N_2_, lyophilized, and ground to a fine powder. Weighed, powdered samples (25 mg) were extracted using 1.5 ml of methanol containing internal standards of 60 ng d_6_-jasmonic acid (HPC Standards GmbH, Cunnersdorf, Germany), 60 ng salicylic acid-d_4_ (Santa Cruz Biotechnology), 60 ng abscisic acid-d_6_ (Toronto Research Chemicals), and 12 ng d_6_-jasmonic acid-isoleucine conjugate (HPC Standards GmbH). A triple-quadruple LC-MS/MS system was used for phytohormone quantification (SCIEX 6500).

### Callose staining, microscopy, and quantification

Seedlings at 2, 7, and 14 dpi were treated with Aniline Blue (0.001%) according to the protocol described by Schenk and Schikora (2015). Colonized and non-colonized seedlings, and control and AtPep1-treated (100 nM) 7 d old seedlings were incubated at room temperature in acetic acid and ethanol (1:3) for decolorization, washed in 150 mM K_2_HPO_4_, and stained with Aniline Blue (0.001%) solution. Slides for observing callose deposition were prepared using 50% glycerol under a Nikon 80i microscope at 358 nm excitation and 460 nm emission. Relative callose intensities were calculated by dividing callose pixels and total pixels using the digital photograph analysis software GIMP (Scalschi *et al.*, 2015).

### Phylogenetic analysis

A total of 123 complete CNGC sequences from seven different plants were selected for construction of the phylogenetic tree, namely *Arabidopsis thaliana*, *Glycine max*, *Medicago truncatula*, *Solanum lycopersicum*, *Zea mays*, *Oryza sativa*, and *Lotus japonicus*. All the amino acid sequences encoding CNGCs were retrieved from previously published reports (Moeder *et al.*, 2011; Nawaz *et al.*, 2014; Charpentier *et al.*, 2016; Chiasson *et al.*, 2017; Moeder and Yoshioka, 2017) and are listed in Supplementary Table S2. A phylogenetic tree was constructed using the MEGA 10 software (https://www.megasoftware.net/; Kumar *et al.*, 2018), in which the sequences were aligned by MUSCLE with default parameters. These aligned sequences were used to build the phylogenetic tree using the maximum likelihood (ML) method and the evolutionary distances were computed using a Jones–Taylor–Thornton matrix-based method with 1000 bootstrap replications.

### Statistical analysis

Statistical differences between treatments were analysed using two-tailed Student’s *t*-tests or one-way ANOVA followed by Tukey’s test in SigmaPlot 13.0. Figures were generated using Origin 6.0 (www.originlab.com).

## Results

### *CNGC19* expression is activated by *P. indica* cell-wall extract

**Fig. 1.**
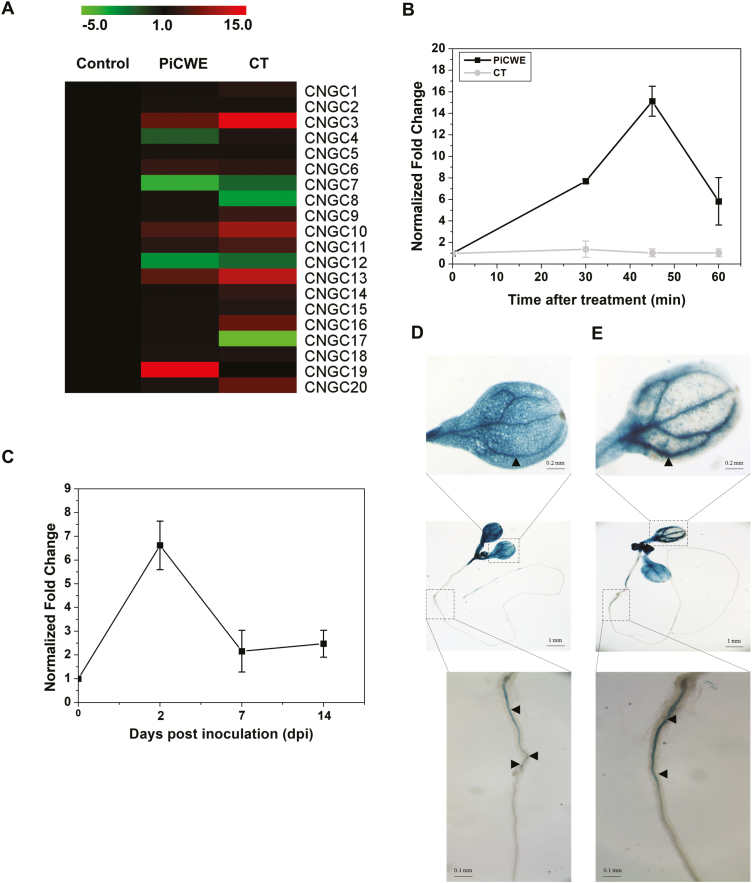
Expression and localization of *CNGC19* in Arabidopsis in response to colonization by *P. indica*. (A) Expression profiling of *CNGC*s in 10 d old seedlings after treatment for 30 min with *P. indica* cell-wall extract (PiCWE) and cellotriose (CT). The heat map represents the fold-change of mRNAs in the treated samples relative to the controls. Data are based on four replicates with six seedling per replicate. Transcripts levels were normalized using *Actin2* mRNA. (B) Expression of *CNGC19* in 10-d-old seedlings at different times after treatment with either 50 µl PiCWE or 10 µM CT. (C) *CNGC19* expression in Arabidopsis seedlings co-cultivated with *P. indica* on 1× PNM. In (A, B), transcripts levels were normalized using *Actin2* mRNA and are presented as the fold-change relative to expression at time zero, which was set as 1. Data are means (±SE) of four replicates, each consisting of six seedlings. (D, E) *CNGC19* promoter activity in roots and leaf vasculature upon *P. indica* colonization at (D) 2 d post-inoculation (dpi) and (E) 7 dpi. Seedlings expressing *ProCNGC19::GUS* were co-cultivated with *P. indica* on 1× PNM. Arrows indicate *ProCNGC19::GUS* expression.

To identify the role of CNGCs in the perception of *P. indica* by Arabidopsis, we applied either crude cell-wall extract (PiCWE) or its identified active elicitor cellotriose (CT) to Arabidopsis seedlings for 30 min (Fig. 1A). Upon treatment with PiCWE, five CNGCs were found to be induced in Arabidopsis roots, namely *CNGC19* (14.3-fold increase), *CNGC3* (6.22-fold), *CNGC13* (5.9-fold), *CNGC10* (5-fold), and *CNGC6* (3.7-fold). In response to CT, nine CNGCs were induced, with the highest expression being found for *CNGC3* (17.5-fold); however, *CNGC19* was not induced by CT. Since it was the highest expressed upon PiCWE treatment and the only transcript specifically induced by PiCWE but not by CT, we hypothesized that *CNGC19* was induced by unidentified elicitors. We examined the patterns of *CNGC19* expression in wild-type (WT) roots and found that it increased from 10–45 min in response to PiCWE but there was no response to CT (Fig. 1B). We then examined roots of plants co-cultivated with *P. indica*, and found that expression of *CNGC19* was increased by 7-fold at 2 d post-colonization, before returning to the basal level at later time-points (Fig. 1C), thus indicating a potential role in the colonization process. To identify the tissue-specific expression pattern of *CNGC19* in Arabidopsis after colonization by *P. indica*, we used *ProCNGC19::GUS*. *CNGC19* promoter activity was observed in the root primordia and primary vasculature at 2 dpi and 7 dpi (Fig. 1D, E). Interestingly, we also observed a systemic expression of *CNGC19* in the leaf vasculature at the same time. The results therefore indicated that *CNGC19* is an early-activated gene that is expressed in the vasculature, and it is induced in the roots by unidentified components in PiCWE and systemically in leaves.

### The growth of *cngc19* mutants is inhibited by *P. indica*

**Fig. 2.**
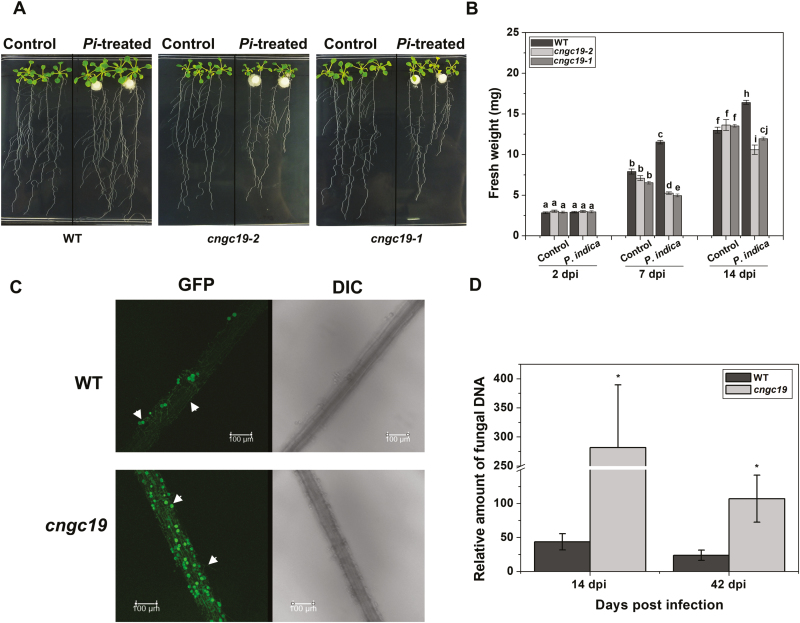
Effects of *P. indica* colonization on Arabidopsis *cngc19* mutants. (A) Representative images of the wild-type (WT), and the *cngc19-2* and *cngc19-1* lines after co-cultivation with *P. indica* (*Pi*-treated) for 14 d compared with non-inoculated controls. (B) Fresh weights of the WT, *cngc19-2*, and *cngc19-1* at 2–14 d post-inoculation (dpi) with *P. indica* compared with non-inoculated controls. Data are means (±SE), *n*=20 seedlings. Different letters indicate significant differences among the means as determined using one-way ANOVA and a *post hoc* Tukey test (*P*≤0.05). (C) Colonization patterns of *P. indica* on the WT and *cngc19-2* as determined by confocal microscopy. GFP-tagged *P. indica* was visualized at 14 dpi and arrows indicate chlamydospores and hyphae. DIC, differential interference contrast images. (D) Quantification of *P. indica* colonization in the WT and *cngc19-2* mutant grown on plates (14 dpi) and in soil (42 dpi). The relative fungal colonization was calculated by subtracting the *C*_T_ values of *P. indica Tef1* from the *C*_T_ values of Arabidopsis *Actin2*. Data are means (±SE) of four replicates, each of which consisted of the combined roots of six seedlings. Significant differences were determined using two-tailed Student’s *t*-tests (*P≤0.05).

To identify the functional role of CNGC19 in the promotion of growth induced by *P. indica*, we utilized the *cngc19-2* and *cngc19-1* mutant T-DNA lines (Meena *et al.*, 2019). In contrast to the WT plants, we observed no growth promotion in the *cngc19* mutants in response to *P. indica* for parameters such as fresh weight (Fig. 2A, B) and root length (Supplementary Fig. S1) when plants were grown in culture medium. Indeed, the mutants showed growth inhibition at 7 dpi and 14 dpi compared to the WT (Fig. 2B). We found similar effects when we repeated the experiment with plants grown in soil, with the growth of the mutants being strongly inhibited at 42 dpi whereas growth promotion was observed in the WT in response to *P. indica* (Supplementary Fig. S2A–C).

### Colonization by *P. indica* is enhanced in *cngc19* roots

We tested the hypothesis that the reduced growth in *cngc19* mutants upon *P. indica* inoculation was due to enhanced colonization. The roots of the WT and *cngc19-2* were co-cultivated with GFP-tagged *P. indica* and were used for microscopic analyses. We observed no differences between *P. indica* colonization in the WT and *cngc19-2* at 2 dpi and 7 dpi (Supplementary Fig. S3); however, at 14 dpi *cngc19* roots had increased colonization compared to the WT and clumps of fungal mycelia and spores could be observed (Fig. 2C). This was supported by quantification of the relative fungal DNA content, which increased at 14 dpi (grown in medium) and 42 dpi (grown in soil) in *cngc19-2* roots relative to the WT (Fig. 2D). Thus, the loss-of-function of CNGC19 resulted in increased colonization and loss of controlled *P. indica* growth in the plant–fungal interaction at the post-establishment phase.

### The CNGC19 channel is involved in PiCWE-mediated elevation of cytosolic Ca^2+^

**Fig. 3.**
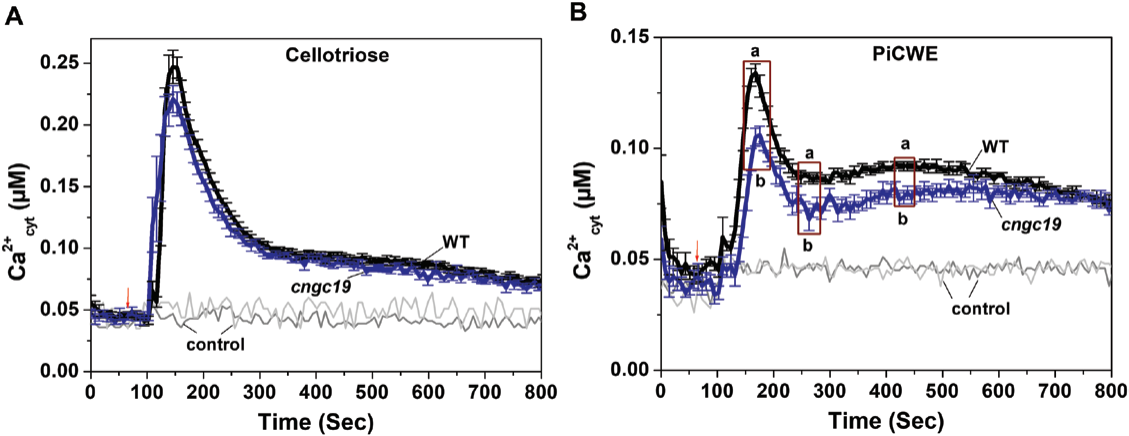
Concentrations of cytosolic calcium (Ca^2+^_cyt_) in Arabidopsis in response to treatment with *P. indica*-related elicitors. Roots of transformed 10-d-old seedlings of the wild-type (WT) and *cngc19* expressing cytosolic apoaequorin were treated with (A) cellotriose (10 µM) or (B) *P. indica* cell-wall extract (50 µl). Data are means (±SE), *n*=5. The experiment was repeated three times with similar results and the data from one experiment are shown. Water was used as the control and gave background readings in the WT and *cngc19*. The arrows indicate the time of treatment with the elicitors. Different letters indicate significant differences between the WT and *cngc19* during the selected periods enclosed in the boxes, as determined using one-way ANOVA and a *post hoc* Tukey test (*P*≤0.001).

CNGC19 is a plasma membrane-localized Ca^2+^-permeable channel (Meena *et al.*, 2019) and we therefore hypothesized that it could be involved in generating the elevation in cytosolic Ca^2+^ in response to PiCWE. We used the WT*::aeq* and *cngc19::aeq* lines to examine intracellular Ca^2+^_cyt_ upon application of PiCWE and CT to the roots. The substrate of CT, cellobiose (CB), was also used as an unrelated elicitor control. Both CT (Fig. 3A) and cellobiose (Supplementary Fig. S4) induced elevation of Ca^2+^_cyt_ in the WT*::aeq* and *cngc19* roots at similar levels. When PiCWE was added, the elevation in Ca^2+^_cyt_ was reduced in the *cngc19::aeq* line relative to the WT*::aeq* (Fig. 3B), both in the initial peak and for several minutes thereafter. These results suggest that CNGC19 is a crucial channel that is involved in sensing as yet unidentified elicitors in PiCWE and in activating the elevation of Ca^2+^_cyt_. 

### Callose deposition in response to *P. indica* colonization is delayed in *cngc19*

**Fig. 4.**
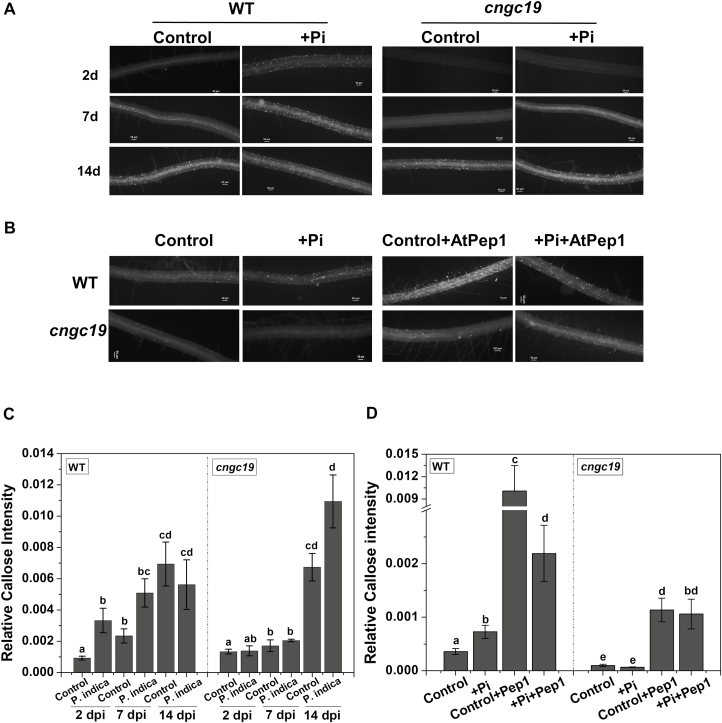
Patterns of callose deposition in the roots of the Arabidopsis wild-type and the *cngc19* mutant in response to colonization by *P. indica*. (A) Representative images of callose deposition in roots of non-inoculated controls and in roots colonized with *P. indica* (+Pi) at 2–14 d post-inoculation (dpi) in the wild-type (WT) and the *cngc19-2* mutant. (B) Representative images of callose deposition in roots of non-inoculated controls and in roots colonized with *P. indica* for the WT and *cngc19-2* plants with or without AtPep1 treatment (100 nM) applied for 24 h at 2 dpi. (C) Relative callolose intensity of the roots shown in (A) as determined from staining for non-inoculated controls and for roots colonized by *P. indica* for WT and *cngc19-2* seedlings at 2–14 dpi. The intensity was expressed as the number of fluorescent callose-corresponding pixels relative to that of the total number of pixels. Data are is means (±SE) of *n*=20 seedlings. (D) Relative callose intensity in roots shown in (B). Data are means (±SE) of *n*=15 seedlings. In (C, D), different letters indicate significant differences among the different treatments, as determined by one-way ANOVA and a *post hoc* Tukey Test (*P*≤0.001).

Increased callose deposition has been reported in Arabidopsis roots colonized by *P. indica* and indicates the activation of MTI (Jacobs *et al.*, 2011). In the WT plants, callose deposition was increased at 2 dpi and 7 dpi and then remained unchanged at 14 dpi during the established colonization phase (Fig. 4A, C). In contrast, in the *cngc19-2* mutant the callose deposition was unaltered compared to the control at both 2 dpi and 7 dpi, indicating a reduced defense in these plants at these early stages. Callose deposition was not induced in *cngc19-2* until 14 dpi. Thus, plant defense was lowered in the *cngc19* mutant during the initial stages of the plant–fungal interaction, leading to increased colonization.

### CNGC19-mediated basal immunity upon perception of *P. indica* is dependent on AtPep-PEPR

**Fig. 5.**
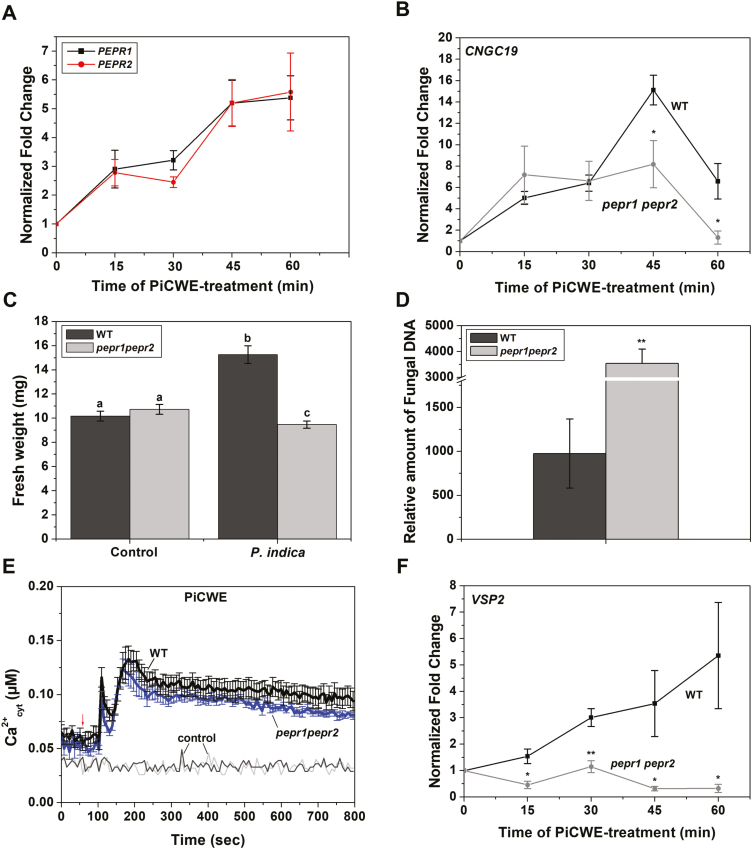
The roles of *PEPR1* and *PEPR2* in the Arabidopsis–*P. indica* interaction. Expression of (A) *PEPR1* and *PEPR2* in wild-type (WT) and (B) expression of *CNGC19* in the *pepr1 pepr2* double-mutant in response to treatment with *P. indica* cell-wall extract (PiCWE) in 10-d-old seedlings. Transcripts levels were normalized to *AtActin2* mRNA and the fold-change in expression is relative to the value at time zero, which was set as 1. Data are means (±SE) of four replicates, each of which consisted of six seedlings. Significant differences were determined using two-tailed Student’s *t*-test (*P<0.05). (C) Effects of *P. indica* colonization on the fresh weight of the WT and the *pepr1 pepr2* double-mutant in non-inoculated controls in response to colonization by *P. indica* at 14 d post-inoculation (dpi). Data are means (±SE), *n*=30. Different letters indicate significant differences among the different treatments, as determined using one-way ANOVA and a *post hoc* Tukey Test (*P*≤0.001). (D) Effect of *P. indica* colonization on fungal colonization in the WT and the *pepr1 pepr2* double-mutant. Plants were co-cultivated with or without fungal discs on 1× PNM agar plates and the roots were harvested at 14 dpi. The relative fungal colonization was calculated by subtracting the *C*_T_ values of *P. indica Tef1* from the *C*_T_ values of Arabidopsis *Actin2*. Data are means (±SE) of four replicates, with six seedlings per replicate. The significant difference was determined using a two-tailed Student’s *t*-test (**P≤0.005). (E) Response of cytosolic calcium (Ca^2+^_cyt_) to treatment with *P. indica* cell-wall extract (PiCWE, 50 µl) in roots of transformed 10-d-old seedlings of the WT and *pepr1 pepr2* expressing cytosolic apoaequorin. Data are means (±SE), *n*=5. The experiment was repeated three times with similar results and the data from one experiment are shown. Water was used as the control and gave background readings in the WT and *pepr1 pepr2*. The arrow indicates the time of treatment with the elicitor. (F) Expression of the defense-related gene *VSP2* in response to treatment with PiCWE in roots of transformed 10-d-old seedlings of the WT and *pepr1 pepr2* double-mutant expressing cytosolic apoaequorin. Transcripts levels were normalized to *AtActin2* mRNA and the fold-change in expression is relative to the value at time zero, which was set as 1. Data are means (±SE), *n*=4.) Significant differences were determined using two-tailed Student’s *t*-test (*P≤0.05; **P≤0.005).

Plants roots encounter damage-associated molecular patterns (DAMPs) upon microbial invasion (Boller and Felix, 2009; Albert, 2013). In Arabidopsis, a family of endogenous elicitor peptides referred to as AtPeps acts as DAMPs (Huffaker and Ryan, 2007; Bartels *et al.*, 2013) and the plasma membrane Pep-receptors PEPR1 and PEPR2 perceive them (Yamaguchi *et al*., 2006, 2010; Krol *et al.*, 2010). We tested the possibility that *P. indica* may suppress AtPep-induced defense, and the role of CNGC19 in such a process. We found that callose deposition induced by application of AtPep1 was suppressed in WT seedlings inoculated with *P. indica* at 2 dpi (Fig. 4B, D). For the *cngc19* mutant, the AtPep1-induced deposition of callose was constitutively lower than that of the WT for all the treatments, and it was not suppressed by colonization by *P. indica* (Fig. 4D). Thus, the results indicated that CNGC19 was also crucial for *P. indica*-mediated suppression of DAMP-triggered immunity. The Pep-receptors PEPR1 and PEPR2 have a putative guanyl cyclase domain that generates cyclic nucleotides and they are upstream of CNGC2 (Ma *et al.*, 2012). In order to determine the role of PEPR1 and PEPR2 in the Arabidopsis–*P. indica* association, we examined their expression. In WT seedlings, expression of *PEPR1* and *PEPR1* was found to be induced by PiCWE treatment (up to 3.5-fold; Fig. 5A) and by *P. indica* colonization (up to 6-fold; Supplementary Fig. S5A). To examine the dependency of CNGC19 activation on PEPR, we measured the expression of *CNGC19* in the *pepr1 pepr2* background in response to treatment with PiCWE, and found that it was reduced at 45 min and 60 min relative to the WT (Fig. 5B). We then examined the role of PEPRs in *P.indica*-induced growth promotion. Colonization by *P. indica* was relatively high in *pepr1 pepr2*, and instead of growth promotion, inhibition was observed both on plates (Fig. 5C, D) and in soil (Supplementary Fig. S6A, B). However, elevation of Ca^2+^_cyt_ was unaltered when compared to the WT in *pepr1 pepr2::aeq* upon treatment with PiCWE (Fig. 5E) and CT (Supplementary Fig. S5B). PEPR signaling contributes to the JA signaling pathway upon herbivory (Klauser *et al.*, 2015; Meena *et al.*, 2019). The level of the JA marker *VSP2* was found to be reduced in *pepr1 pepr2* upon both treatment with PiCWE and colonization by *P. indica* (Fig. 5E, Supplementary S5C), indicating its function downstream of CNGC19 via jasmonate signaling.

### Loss-of-function of CNGC19 down-regulates jasmonate biosynthesis upon *P. indica* colonization

**Fig. 6.**
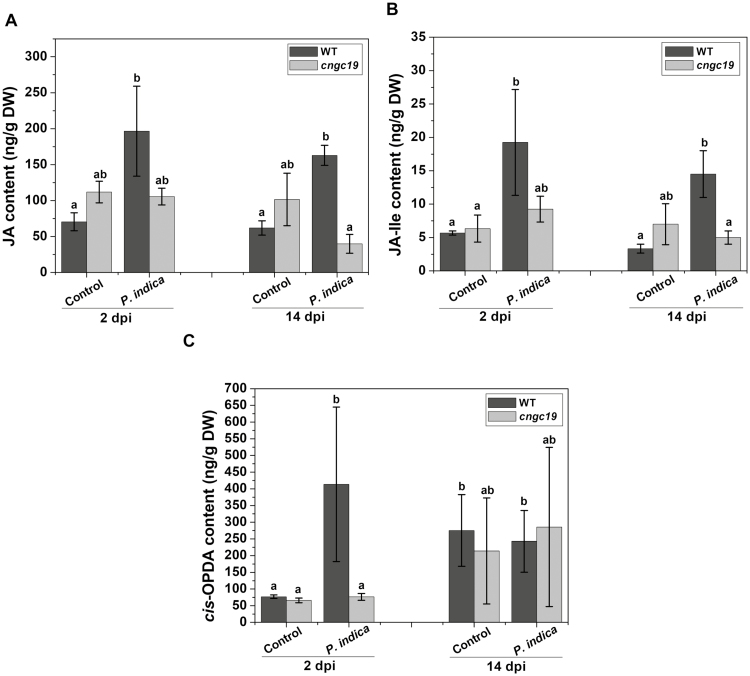
Effects of *P. indica* colonization on levels of phytohormones in seedlings of the Arabidopsis wild-type (WT) and the *cngc19* mutant. (A) Jasmonates (JA), (B) the JA bioactive form (+)jasmonoyl isoleucine (JA-ILE), and (C) the JA precursor *cis*-(+)-12-oxo-phytodienoic acid (*cis*-OPDA) at 2 d post-inoculation (dpi) and 14 dpi. Data are means (±SE) of three replicates, each of which consisted of 40 seedlings. Different letters indicate signiﬁcant differences between the WT and *cngc19-2* plants at both time-points as determined using one-way ANOVA and a *post hoc* Tukey Test (*P*≤0.001).

*Piriformospora indica* activates the JA signaling pathway in Arabidopsis, and this is crucial for colonization and for balancing the beneficial interaction between the two organisms (Stein *et al.*, 2008; Vahabi *et al.*, 2015). To identify the role of various phytohormones in *P. indica* colonization in the *cngc19* mutant, we measured their levels at 2 dpi and 14 dpi. The levels of JA and jasmonoyl isoleucine (JA-Ile) were increased in WT plants in response to *P. indica* at 2 dpi and 14 dpi, and *cis*-(+)-12-oxo-phytodienoic acid (*cis*-OPDA) was also strongly increased at 2 dpi (Fig. 6A-C). In contrast, no significant effects of colonization were observed for *cngc19*. The lack of an effect on the levels of *cis*-OPDA, JA, and JA-Ile at 2 dpi may have contributed to uncontrolled colonization in *cngc19* roots. JA-Ile-OH also showed similar trends (Supplementary Fig. S7A), but no changes in ABA and salicylic acid (SA) were observed in response to *P. indica* colonization in either genotype at either time-point (Supplementary Fig. S7B, C).

### Phytohormone- and defense-related genes are down-regulated in *cngc19* during colonization

**Fig. 7.**
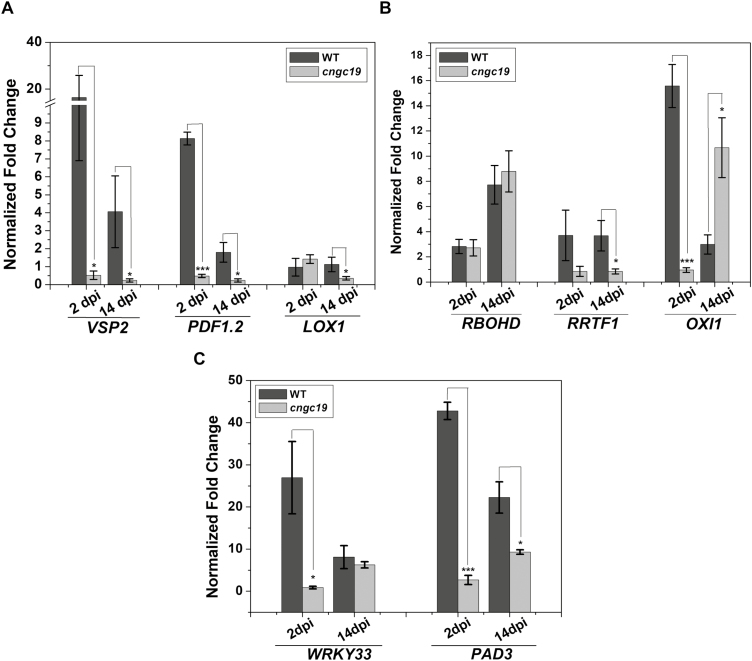
Effects of *P. indica* colonization on expression of genes related to phytohormones, reactive oxygen species (ROS), and defense in seedlings of the Arabidopsis wild-type (WT) and the *cngc19* mutant. Expression of marker genes for (A) jasmonates, (B) ROS, and (C) defense early 2 d post-inoculation (dpi) and 14 dpi. Transcripts levels were normalized to *AtActin2* mRNA and the fold-change in expression is relative to that of the corresponding non-inoculated control, which was set as 1. Data are means (±SE) of four replicates, each of which consisted of six seedlings. Significant differences were determined using two-tailed Student’s *t*-test: *P≤0.05, ***P<0.0001.

Since colonization by *P. indica* was enhanced in the *cngc19* lines, we examined the expression of marker genes of different defense pathways such as those of JA, ROS, and phytoalexin. The JA markers *VSP2*, *PDF1.2*, and *LOX1* were found to be induced by *P. indica* in WT plants at both 2 dpi and 14 dpi compared to non-inoculated controls, whereas these genes were found to be down-regulated in *cngc19* except *LOX1* at 2 dpi (Fig. 7A). The ROS markers *RBOHD*, *RRTF1*, and *OXI1* were selected based on their known functional roles in the Arabidopsis–*P. indica* interaction (Camehl *et al.*, 2011; Matsuo *et al.*, 2015; Johnson *et al.*, 2018). *RRTF1* was found to be up-regulated in the WT but not in *cngc19* (Fig. 7B), *RBOHD* was up-regulated in both the WT and *cngc19* and did not differ between the two, and *OXI1* was strongly up-regulated at 2 dpi in the WT but showed no change in *cngc19* compared to non-inoculated controls. In contrast, at 14 dpi OXI1 was observed to be strongly up-regulated in *cngc19*, which may have been due to increased colonization. In addition, the ROS-related genes *SOD1*, *GSTF8*, and *APX1* were found to be up-regulated in the WT and down-regulated in *cngc19* at 2 dpi (Supplementary Fig. S8), and at 14 dpi *GR1* and *CAT2* were found to be up-regulated in *cngc19* but unaltered in the WT. *WRKY33* and *PAD3*, which are related to phytoalexin biosynthesis, were also found to be up-regulated in the WT but not in *cngc19* at 2 dpi (Fig. 7C). At 14 dpi, *WRKY33* was up-regulated in both the WT and *cngc19* and did not differ between the two, whilst *PAD3* was also up-regulated in both but had a greater increase in the WT. Thus, the loss-of-function of *CNGC19* affected JA-responsive genes, and genes involved in ROS signaling and defense, and was associated with over-colonization by *P. indica* in *cngc19* plants.

### CNGC19-mediated defense signaling in roots acts via indole glucosinolates

**Fig. 8.**
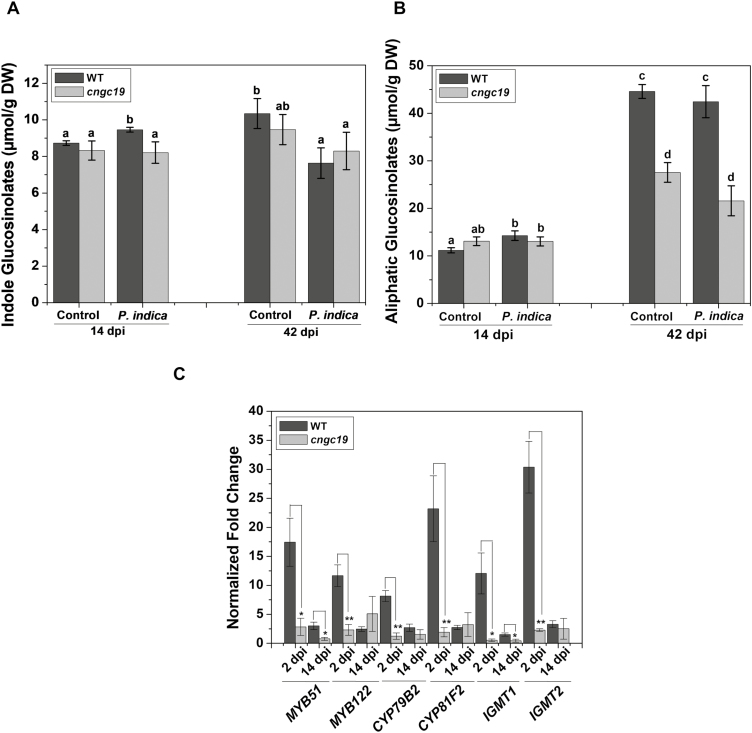
Effects of *P. indica* colonization on glucosinolates in seedlings of the Arabidopsis wild-type (WT) and the *cngc19* mutant. Levels of (A) indole glucosinolates (iGS) and (B) aliphatic glucosinolates in non-inoculated controls and plants colonized by *P. indica* grown either on plates (14 d post-inoculation, dpi) or in soil (42 dpi). Data are means (±SE) of three replicates each consisting of 40 seedlings (14 dpi) or five replicates each consisting of one seedling (42 dpi). Different letters indicate significant differences among the different treatments as determined using one-way ANOVA and a *post hoc* Tukey’s test (*P*≤0.001). C. Relative expression of iGS biosynthesis pathway genes in seedlings grown on plates at 2 dpi and 14 dpi. Transcripts levels were normalized to *AtActin2* mRNA and the fold-change in expression is relative to that of the corresponding non-inoculated control, which was set as 1. Data are means (±SE) of four replicates each consisting of six seedlings. Significant differences were determined using two-tailed Student’s *t*-test (*P≤0.05, **P≤0.005).

*CNGC19* loss-of-function results in constitutively reduced levels of aliphatic GSs in Arabidopsis rosettes, which are crucial for defense against herbivory (Meena *et al.*, 2019). Upon plant–microbe interactions, accumulation of antimicrobial indole glucosinolates (iGSs) and camalexin trigger immunity (Bednarek *et al.*, 2009; Clay *et al.*, 2009; Böhm *et al.*, 2014). The iGS pathway is critical for mutualistic *P. indica* colonization and for uncompromised plant immunity (Nongbri *et al.*, 2012; Lahrmann *et al.*, 2015). We therefore decided to test the effects of mutation in *CNGC19* on iGS levels. We found that the constitutive levels of both iGSs and aliphatic GSs in non-inoculated (control) seedlings were the same in the WT and *cngc19* at 14 dpi (Fig. 8A, B). At the rosette stage in non-inoculated plants grown in soil (42 dpi), the level of iGSs did not differ between the WT and *cngc19* whilst aliphatic GSs were significantly lower in *cngc19*, as also reported by Meena *et al.*, 2019. We then looked at the effects of *P. indica* colonization and found that iGSs were increased in the WT at 14 dpi but were not affected in *cngc19* (Fig. 8A). The levels of aliphatic GSs also showed a similar trend (Fig. 8B). After a prolonged period of colonization by *P. indica* (42 dpi, grown in soil), iGS levels were reduced significantly in WT plants but were unaltered in *cngc19* (Fig. 8A). Aliphatic GS levels did not change at 42 dpi in either of the genotypes (Fig. 8B). We examined the relative expression of key genes related to the iGS biosynthesis pathway and found that they were generally up-regulated in the WT but not in *cngc19* at 2 dpi and 14 dpi (Fig. 8C). The results therefore indicate that CNGC19 plays a crucial role in modulating the content of indole GSs during colonization by *P. indica*.

### Phylogenetic analysis of CNGC19 indicates it has a distinct role in microbial interactions

**Fig. 9.**
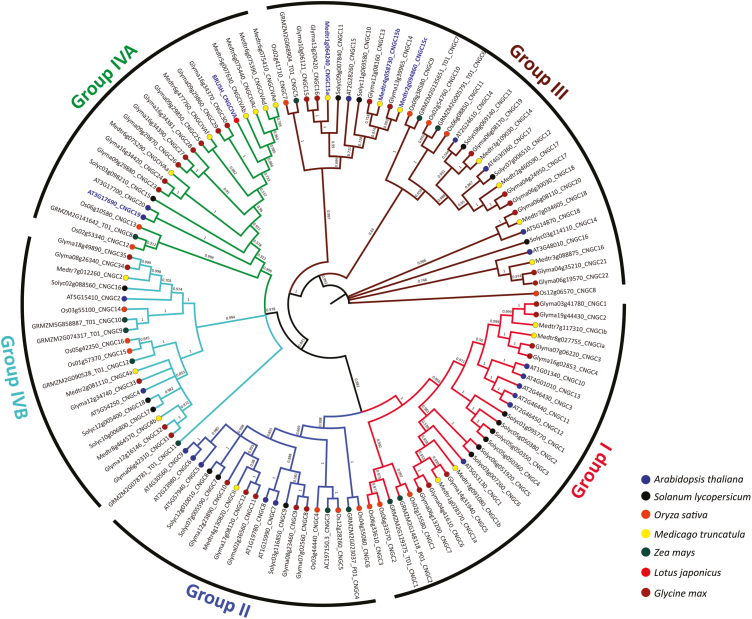
Phylogenetic relationships of AtCNGC19 with homologs from other species. Multiple sequence analysis was performed using the MUSCLE software. A maximum-likelihood unrooted tree was constructed using 123 complete amino acid sequences of CNGCs in the MEGA10 software. The evolutionary relationships were analysed with 1000 bootstrap replicates. Each node is labelled with the gene ID and its previously reported name. Genes examined in the current study are highlighted in blue.

Unlike rhizobial nodulation and mycorrhizal symbiosis, *P. indica* has a broad host range and is a primitive symbiont (Franken *et al.*, 2000). We constructed a phylogenetic tree in order to understand the relationship between AtCNGC19 and its orthologs in other host plants, many of which form symbiotic interactions. Genome-wide analyses in seven different species have identified distinct CNGCs (Mäser *et al.*, 2001; Nawaz *et al.*, 2014; Saand *et al.*, 2015; Charpentier *et al.*, 2016). In our unrooted phylogenetic tree, 123 CNGCs clustered into four different groups (Groups I–IV; Fig. 9). Among these, Group IV was further subdivided into IVA and IVB. AtCNGC19 and AtCNGC20 were clustered into Group IVA with orthologs from different legume plants that form symbiotic interactions, such as *G. max*, *M. truncatula*, and *L. japonicas*. AtCNGC19 was clustered with BRUSH from *L. japonicas*, which plays a crucial role in rhizobial symbiosis by regulating Ca^2+^ fluxes (Chiasson *et al.*, 2017). AtCNGC19 also grouped with OsCNGC13 (its ortholog in *O. sativa*; Moeder and Yoshioka, 2017), which has been shown to be up-regulated by a bacterial pathogen (Nawaz *et al.*, 2014). Nuclear-localized MtCNGC15 has been shown to be critical in generating oscillations in nuclear Ca^2+^ during mycorrhizal symbiosis (Charpentier *et al.*, 2016); however, it was found to be clustered into Group III, which suggests it is evolutionarily divergent from AtCNGC19.

## Discussion

Plant roots interact with both pathogenic and symbiotic microbes and recognize them as a potential threat by activation of basal MAMP-triggered immunity (MTI). Activation of MTI prevents the establishment of pathogens and regulates colonization by symbionts, and thus the process acts as a gatekeeper (Yu *et al.*, 2019*a*). Root MTI efficiently restricts penetration and colonization of the mutualist *P. indica* and prevents over-colonization, with the result that a symbiotic interaction is established (Jacobs *et al.*, 2011). Recognition of *P. indica* cell-wall extract (PiCWE) by Arabidopsis roots induces elevation of cytosolic Ca^2+^ (Ca^2+^_cyt_) and is crucial in activating the symbiotic interaction and in promoting growth (Vadassery *et al.*, 2009; Johnson *et al.*, 2018). The identity of the ion channels responsible for the influx of Ca^2+^ and the activation of signaling is currently unknown. In our present study, we identified *CNGC19* as an early-activated gene (Fig. 1A) and found that it was induced by unidentified components in PiCWE and not by treatment with cellotriose (CT) (Fig. 1A, B). Cyclic nucleotide gated channels (CNGCs) are altered by treatment with PiCWE and glutamate receptor-like channels (GLRs) are altered by treatment with CT in plant roots (Vadassery *et al.*, 2009; Johnson *et al.*, 2018). PiCWE (which contains many elicitors including CT) and CT also differ in their activation of other pathways. Importantly, CT induces a defense pathway comprising ROS accumulation and the expression of its marker gene *RBOHD* (Johnson *et al.*, 2018), whereas PiCWE does not activate this pathway (Vadassery *et al.*, 2009). We found that CNGC19 was crucial for *P. indica*-induced promotion of growth, as its loss-of-function resulted in increased colonization and the complete loss of growth-promotion phenotype (Fig. 2A, B). Importantly, CNGC19 was found to be critical for the generation of the PiCWE-induced elevation of Ca^2+^_cyt_. No other Ca^2+^ channels have yet been implicated in the growth promotion and elevation of Ca^2+^_cyt_ that is induced by *P. indica*. Since PiCWE-induced Ca^2+^ signals were not completely abolished in the *cngc19* mutant (Fig. 3B), they might be controlled by additional genetic interactions between CNGCs and other unknown channels. A leucine‐rich repeat protein mutant, *Piriformospora indica-insensitive12* (*pii12*), has previously been reported to show no promotion of growth when it is associated with *P. indica* (Shahollari *et al.*, 2007). Overall, our results indicate that the PiCWE-activated CNGC19 is a critical Ca^2+^ channel for growth-promotion signaling.

Expression of *CNGC19* in Arabidopsis is associated with salinity stress (Kugler *et al.*, 2009; Oh *et al.*, 2010). We have previously reported that CNGC19 expressed in the leaf vasculature is crucial for Arabidopsis defense against herbivory by *Spodoptera* moths by regulating the spread of Ca^2+^ signals and the levels of jasmonate and aliphatic glucosinolates (Meena *et al.*, 2019). The AtCNGC19 homolog SlCNGC15 in tomato is induced by both salinity stress and *P. indica* colonization (Ghorbani *et al.*, 2019). SlCNGC15 is also associated with disease resistance against the necrotropic fungus *Sclerotinia sclerotiorum* (Saand *et al.*, 2015). *cngc19* and *cngc20* mutants are also more susceptible to infection by *Botrytis cinerea* (Moeder *et al.*, 2011). The receptor kinase BAK1/SERK4 phosphorylates the Ca^2+^-channel complex CNGC20/CNGC19 and has crucial role in pathogen-induced cell death (Yu *et al.*, 2019*b*). The roles of other CNGCs in plant defense have been demonstrated in many studies. AtCNGC2 and AtCNGC4 are known to regulate Ca^2+^-induced PAMP-triggered immunity (Chin *et al.*, 2013; Tian *et al.*, 2019). They act as a heterotetrameric Ca^2+^ channel and are phosphorylated and activated by the kinase BIK1 of the pattern-recognition receptor complex, triggering an increase in the concentration of Ca^2+^_cyt_ (Tian *et al.*, 2019). CNGC2 and CNGC4, which are also known as *dnd1* (*defense no death1*) and *dnd2*, respectively, are involved in the hypersensitivity response and DAMP perception during bacterial infection in Arabidopsis (Ahn, 2007). *cngc11* and *cngc12* are also hypersusceptible to fungal infection (Yoshioka *et al.*, 2001). In apple, overexpression of *MdCNGC1* results in increased susceptibility to fungal infection and in reduced callose deposition when plants are treated with *flg22* and chitosan (Zhang *et al.*, 2018). Interestingly, in our study CNGC2, CNGC4, CNGC11, and CNGC12 were not significantly induced by *P. indica* (Fig. 1A), indicating that a different set of channels is involved in the interaction. The *brush* mutant has been isolated in a screen of an ethyl-methanesulfonate-mutated population for plants defective in symbiotic cell development (Maekawa-Yoshikawa *et al.*, 2009). At 26 °C, *brush* roots are stunted and infection threads in root hairs do not progress into the cortex, resulting in the formation of non-infected nodules. This has been mapped as a gain-of-function CNGC.IVA mutation and it is orthologous to AtCNGC19 and AtCNGC20, resulting in a leaky tetrameric channel (Chiasson *et al.*, 2017). Similarly, nuclear-localized CNGC15 in *Medicago* forms a complex with the potassium-permeable channel DMI1, and is responsible for nuclear Ca^2+^ release upon mycorrhizal symbiosis (Charpentier *et al.*, 2016). Our phylogenetic analysis also indicated that CNGCs of diverse plants that clustered in Groups IV and III are involved in both symbiotic and pathogenic interactions (Fig. 9). Thus, activation of Ca^2+^ channels belonging to the CNGC family seems to be a conserved element in symbiotic interactions.

**Fig. 10.**
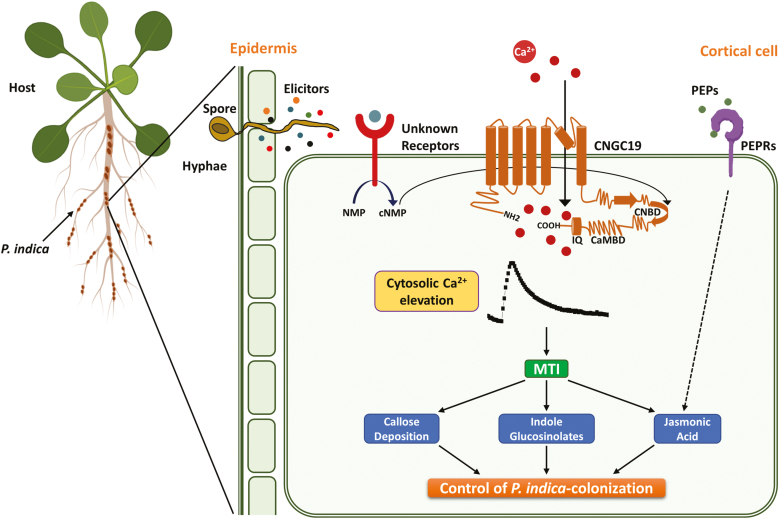
Schematic model of the role of CNGC19 in the interaction between Arabidopsis and *P. indica*. Upon perception of *P. indica* cell wall-associated elicitor(s), as yet unknown receptors are activated in the root and lead to activation of CNGC19, which elevates the level of cytosolic calcium. This in turn modulates downstream systemic and defense-related pathways of MAMP-triggered immunity (MTI), such as callose deposition, glucosinolate biosynthesis, and jasmonic acid signaling. AtPep-PEPR signaling functions downstream of CNGC19 via jasmonate signaling. The activation of these pathways synergistically leads to maintenance of the *P. indica* colonization at a level that provides a mutualistic association between the plant and the fungus.

Increased *P. indica* colonization in the *cngc19* mutants (Fig. 2) indicated that the normal functioning of CNGC19 is crucial for maintaining controlled colonization, and suggests that it has a role in MTI. Plants deposit callose (β1, 3-glucan) into cell walls upon microbial invasion as a part of MTI (Thordal-Christensen, 2003; Nürnberger and Lipka, 2005). Colonization by *P. indica* is known to induce callose deposition and additional exposure to the elicitor *flg22* does not increase callose because of *P. indica*-mediated suppression of late MTI (Jacobs *et al.*, 2011). Thus, the suppression of callose deposition is required for progression of *P. indica* colonization. We found that callose deposition was initially unaltered in *cngc19* (Fig. 4A, C), indicating that the plant defense was lowered and hence led to increased colonization and pathogen-like growth. Plants activate robust MAMP perception and subsequent MTI by the action of phytohormones such as salicylic acid, ethylene, and jasmonate to regulate *P. indica* colonization. It is known that colonization increases JA/JA-Ile levels in co-cultivated plants with the result that plant defense responses are altered and tolerances to pathogenic microbes and root herbivory are improved (Vahabi *et al*., 2013, 2015; Lahrmann *et al.*, 2015; Cosme *et al.*, 2016). It has also been reported that JA signaling is required to suppress late MTI in order to facilitate the progression of *P. indica* colonization at the late biotrophic stage (Jacobs *et al.*, 2011). It is known that the *jasmonate insensitive1-1* (*jin1-1*) and *jasmonate resistant1-1* (*jar1-1*) mutants show no promotion of growth upon *P. indica* colonization (Jacobs *et al.*, 2011). Loss-of-function of *CNGC19* down-regulated jasmonate biosynthesis and JA-responsive genes upon *P. indica* colonization (Fig. 6 and 7A). This suggests that CNGC19-mediated signaling leads to the activation of JA/JA-Ile signaling (Fig. 10), as has also been observed for defense against *Spodoptera* herbivory in Arabidopsis leaves (Meena *et al.*, 2019). In our study, the *cngc19* mutants also displayed reduced expression of early and late MTI-related genes involved in phytohormone, ROS, and secondary metabolite pathways (Fig. 7A–C), which contributed to unbalancing the mutualistic relationship between the fungus and host plant (Fig. 10). We also found that H_2_O_2_-induced *Oxidative Signal Inducible1* (*OXI1*) kinase was reduced in *cngc19* plants at an early stage of colonization (Fig. 7B). The *oxi1* mutant together with the *agc2-2* (OXI1 kinase homolog) and *pdk1.1 pdk1.2* (3-PHOSPHOINOSITIDE-DEPENDENT PROTEIN KINASE1) mutants also have reduced growth upon *P. indica* colonization (Camehl *et al.*, 2011). In addition, we also found that genes related to the antioxidant system were altered at early and late stages of *P. indica* colonization, namely *SOD1*, *GSTF8*, *GR1*, *CAT2*, and *APX1* (Supplementary Fig. S8). Thus, CNGC19 is critical for MTI responses in Arabidopsis roots (Fig. 10).

Damage-associated molecular patterns (DAMPs) are recognized by leucine-rich repeat (LRR)-like receptors, which activate downstream signaling. AtPep1 is perceived as a stronger danger signal by Arabidopsis roots than the MAMP-like bacterial *flg22* or chitin, and hence AtPep-PEPR signaling is a major component of surveillance in the roots (Poncini *et al.*, 2017). We have previously identified that CNGC19 is involved in AtPep1-induced elevation of Ca^2+^_cyt_ (Meena *et al.*, 2019). Pep-induced PEPR signaling further intensifies the plant defense response together with MTI (Ross *et al.*, 2014). Upon addition of AtPep1, *P. indica*-mediated suppression of callose occurred in the WT but not in the *cngc19* mutant (Fig. 4). *pepr1 pepr2* double-mutants also showed a growth inhibition phenotype (Fig. 5C), but these genes are not involved in the PiCWE-induced elevation of Ca^2+^_cyt_ and instead might be acting via the JA pathway (Fig. 5E, F). PEPR signaling maintains basal immunity by regulating JA and SA signaling, locally and systemically (Ross *et al.*, 2014; Yamada *et al.*, 2016), which agrees with the down-regulation of the JA-responsive gene *VSP2* that we observed upon *P. indica* colonization in the *pepr1pepr2* mutant (Fig. 5). Thus, PEPR signaling works downstream of CNGC19, contributes to the JA pathway, and may interact with unknown receptors and kinases for modulating downstream targets.

It is known that *cngc19* mutants are constitutively deficient in aliphatic glucosinolate accumulation and that they hyperaccumulate its precursor, methionine (Meena *et al.*, 2019). CNGC19 modulates aliphatic glucosinolate biosynthesis in tandem with BRANCHED-CHAIN AMINO ACID TRANSAMINASE4 (BCAT4), which is involved in the chain elongation pathway of metionine-derived glucosinolates (Meena *et al.*, 2019). However, this phenotype appeared to be age-dependent as it was absent in the seedlings that we studied, and upon *P. indica* colonization we found that it was the activation of iGS that was important (Fig. 8A). Thus, regulation of glucosinolates by CNGC19 is age- and stimuli-dependent. The indolic glucosinolate pathway plays a major role in the growth restriction of *P. indica* (Lahrmann *et al.*, 2015). The loss-of-function mutants *cyp79b2*/*3* and *cyp81f2*, which are genes involved in iGS biosynthesis, exhibit growth inhibition upon *P. indica* colonization similar to what we observed in *cngc19* (Nongbri *et al.*, 2012; Lahrmann *et al.*, 2015). Cytochrome P450 enzymes (*CYP79B2*, *CYP79B3*, *CYP81F2*) and transcription regulators of iGS (*MYB51*, *MYB122*, *WRKY33*) and other iGS-related genes (*IGMT1*, *IGMT2*, *PAD3*, *PEN2*) have previously been observed to be stimulated during the interaction with *P. indica* (Jacobs *et al.*, 2011; Nongbri *et al.*, 2012; Lahrmann *et al.*, 2015; Peskan-Berghöfer *et al*., 2015). It has also been shown that genes related to the iGS biosynthesis pathway are essential for callose deposition (Clay *et al.*, 2009). In our study, such genes were found to be down-regulated upon *P. indica* colonization in *cngc19* (Fig. 8), and the levels of iGS were increased at 14 dpi and reduced at 42 dpi in colonized WT plants, but were unaltered in *cngc19*. The activation of iGS biosynthesis might occur via activation of CNGC19 in the colonized plants. Some other iGS-related mutants (*myb34/51/122, pen2-1*) have also been shown to have higher levels of *P. indica* colonization (Jacobs *et al.*, 2011; Nongbri *et al.*, 2012; Lahrmann *et al.*, 2015). In addition, the mutant of a β‐glucosidase (ΔPYK10), which is involved in hydrolysing iGS, has also been observed to have no growth promotion upon *P. indica* colonization (Sherameti *et al.*, 2008; Nakano *et al.*, 2017). All these findings place CNGC19 as an upstream element in iGS activation upon *P.indica* colonization.

In conclusion, our results indicate that CNGC19 is activated by as yet unidentified elicitors in the cell-wall extract of *P.indica*. The CNGC19-mediated pathway affects the basal immunity and the levels of phytohormones and glucosinolates in Arabidopsis upon colonization by *P.indica*, and subsequently affects the growth of the plants. These events do not occur in the *cngc19* mutants, and this leads to over-colonization and detrimental effects on plant health and growth (Fig. 10). Thus, CNGC19 plays a central role as a gatekeeper during colonization by *P. indica*, maintaining a robust innate immunity that ensures that the interaction is mutualistic.

## Supplementary data

Supplementary data are available at *JXB* online.

Table S1. Primers used in this study.

Table S2. Amino acid sequences of CNGCs used for constructing the phylogenetic tree.

Fig. S1. Root lengths of WT and *cngc19-2* seedlings co-cultivated with *P. indica*.

Fig. S2. Growth of WT and *cngc19* seedlings co-cultivated with *P. indica* in soil.

Fig. S3. Colonization patterns of *P. indica* in WT and *cngc19* roots.

Fig. S4. Cytosolic Ca^2+^ levels in response to treatment with *P. indica* elicitors.

Fig. S5. Role of PEPR signaling in the Arabidopsis–*P. indica* interaction.

Fig. S6. Growth effects of *P. indica* colonization in the *pepr1 pepr2* double-mutant.

Fig. S7. Phytohormone levels in WT and *cngc19-2* seedlings during *P. indica* colonization.

Fig. S8. Relative gene expression of several ROS-related genes in WT and *cngc19* seedlings after *P. indica* inoculation.

eraa028_suppl_supplementary_table_S1_figures_S1_S8

eraa028_suppl_supplementary_table_S2
