# Supplementary material for: Calcium channel CNGC19 mediates basal defense signaling to regulate colonization by Piriformospora indica in Arabidopsis roots
Source: J Exp Bot. 2020 Jan 20;71(9):2752–68. doi: 10.1093/jxb/eraa028 (PMC7210775; doi:10.1093/jxb/eraa028)
Supplement: eraa028_suppl_supplementary_table_S1_figures_S1_S8 [file eraa028_suppl_supplementary_table_s1_figures_s1_s8.pdf]

Supplementary Table 1

Gene	Forward primer	Reverse primer
AtCNGC1	CCCAAGCCTCGGTGCTACAA	GGCATTCGTTCCGGCATCCT
AtCNGC2	TCTTCAGGTGGATTGGACTGT	TCCACCGTTGATTTGGAGGT
AtCNGC3	GACGACCCGCGTCGCTAGTA	AGCACCAGCCCAAGCAGTTT
AtCNGC4	CCTAAACCCAATCCCGACGA	TTCTCTATTAGACTTGTGACCACTT
AtCNGC5	TGAAACCGTGTAGCCATCTTG	GAGCCTTCCAAGATCACTTGCT
AtCNGC6	GCGTTAGTCCGAAGAGTTCCA	CCCTCACGAACCAAGTACGA
AtCNGC7	GCTTTTGCCGAAGGAGCTTG	GCTCCAGTTATGTGGCTTGC
AtCNGC8	ACCTCAGATTGCTGTGTGGAA	ATAGCGCCTGTTTTGTTGGT
AtCNGC9	GAGCGGTTGAAGCCGTGTCT	ACTCTCAAGCCGACCACGGA
AtCNGC10	TTGGCATCTTCACTGATGCTCT	ATTCTGTCCCAATGCACTCA
AtCNGC11	GAGAAGAACCAAAGGCACCG	AAGGCACCTTTTTGAGCAACT
AtCNGC12	TCTTCTCCCTTCTTGCATGG	AAGAATACTCTGACTCAACAAGGA
AtCNGC13	CGCAACAATCGTGTAAGGTTCA	AGTCCTCTACGAACATTCTTCAA
AtCNGC14	TGTGAAACCGCATTTCGCTG	CTCATCCGGTTTAGGCAGCA
AtCNGC15	CTGTGAGATTTCAAGAAGATCAAGA	GAGAGTACTTTGGCTTTCAAGAAC
AtCNGC16	GTTATGGACCGCCGTGAAATC	GGTTCTGGTGAGCTGCGTAT
AtCNGC17	TTACCCACTGATCTTCGCCGT	AGAGAAGAAACCAGGCGCTCA
AtCNGC18	TCCGTGCCCTCTCTGAAGTA	CCCATGCTCTCCATTGATGTG
AtCNGC19	CCTTATTGGAGGTTACGAGCA	CTACCAAACCAAACATCATCATCA
AtCNGC20	GATTCAAGTGGCGTGGAGAT	CTATGAGCAGTCTTGAGCTTTG
AtRRTF1	ATGCGGCTCGAGCTTATGAT	CTCCGTTCCATTGCTCTGCT
AtRBOHD	CCGAAGGTCCTTATCGACGG	GTTCTCAATGTCGCTGTCGC
AtOXI1	GGGGAATGGGTTAAGGGGTT	CCCATTTGAAGCCAAGGTCC
AtPAD3	AAGAGATCATCAATGGGGAAGAA	ACTCCAACTGGATCATCACTTTTTA
PiTef1	TCGTCGCTGTCAACAAGATG	GAGGGCTCGAGCATGTTGT
AtMYB51	CGATGAATCCCACGTGACCA	ACGAAACAACACACTTCAGACC
AtMYB122	TCAGAAGGTGAGAGGTCAACG	TCGTATAAACACGGGTCGGG
AtCYP79B2	CCGCCGATGAAATCAAACCC	CGAGTCTCTCTTTCCCGACG

AtCYP81F2	CCTGAGCGGTTCGAAGACC	GCCATTCCAGGATTCTCTGTCA
AtIGMT1	GTTTGCCAAGCTTATCACTGCT	TGCTAGATTGCTTGAAGACAAAGAC
AtIGMT2	TGCCAAGCTTATCACTGCTGG	TGCCAAGCTTATCACTGCTGG
AtVSP2	AAACCGTGTGCAAAGAGGCT	CGGTCTTCTCTGTTCCGTATCC
AtPDF1.2	CTTTGCTGCTTTCGACGCAC	TGATCCATGTTTGGCTCCTTCA
AtLOX1	ACGCCTGGTGAAGGTGTCTA	TGTGTTTGCATCCAGTGGCTA
AtWRKY33	CTCGTGGTAGCGGTTACGCC	CCTTTGCTCTAGAGAATCCACC
AtPEPR1	GCTACATTGCACCAGAAAACG	CACCGCTCTCTTCCTCGTAA
AtPEPR2	ATTGCACCAGAAAATGCGTACA	GTCCAGTGCTCTCTTTCCTGT
AtSOD1	TTTTGGACCTCGTGGGCTAT	TCGCCATTGTTACTTGGCCT
AtGSTF8	GTTGGGTAAAATCCGGCTCAG	AGAGTAGCGTCAAGACGAAGAA
AtGR1	TCAGTTCTGTACTCGCATATGGT	TGCAACTTCTCCAGGAGCTTT
AtCAT2	ACAGTGGCCACATTTGTGTA	ACCCCAAAATGTCAAAACCAAGTT
AtAPX1	TTCTGTTCTGTGGTTCGATATTAGC	AAAGTTCCAGCAGAGTGCCA
AtActin	TCAGATGCCCAGAAGTCTTGTTC	GTGGATTCCAGCAGCTTCCA
ProCNGC19	ACGCGTCGACGAGAGAAGATAAAGAGA AAATGGCA	CGGGATCCTCAGAGGCAGAAACTCT GGAAGTGTG

Figure S1

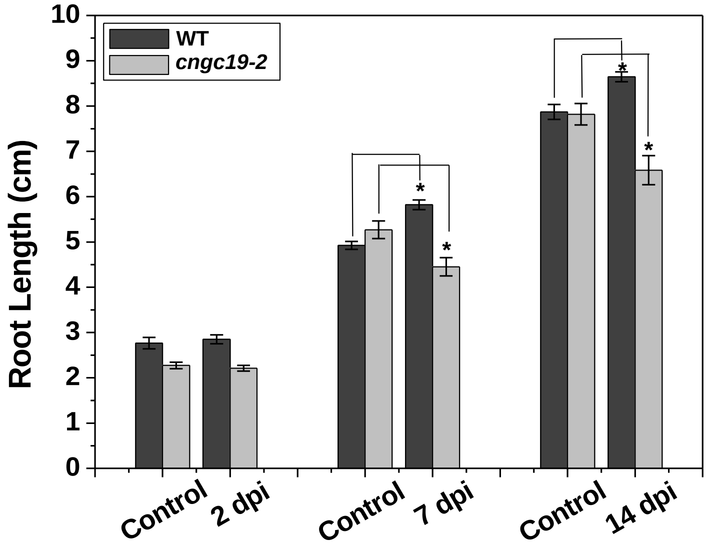

Figure S1. Root length of WT and *cngc19-2* seedlings co-cultivated with *P. indica*

Arabidopsis seedlings were co-cultivated with *P. indica* and the root lengths were measured at 2, 7 and 14 days post inoculation (dpi) in WT (black) and *cngc19-2* (grey) lines. Data shown is Mean ± SE, N=25. Asterisk (*) indicates a significant difference between control and co-cultivated plants, calculated by 2 tailed Student's *t*-test at P≤ 0.05.

Figure S2

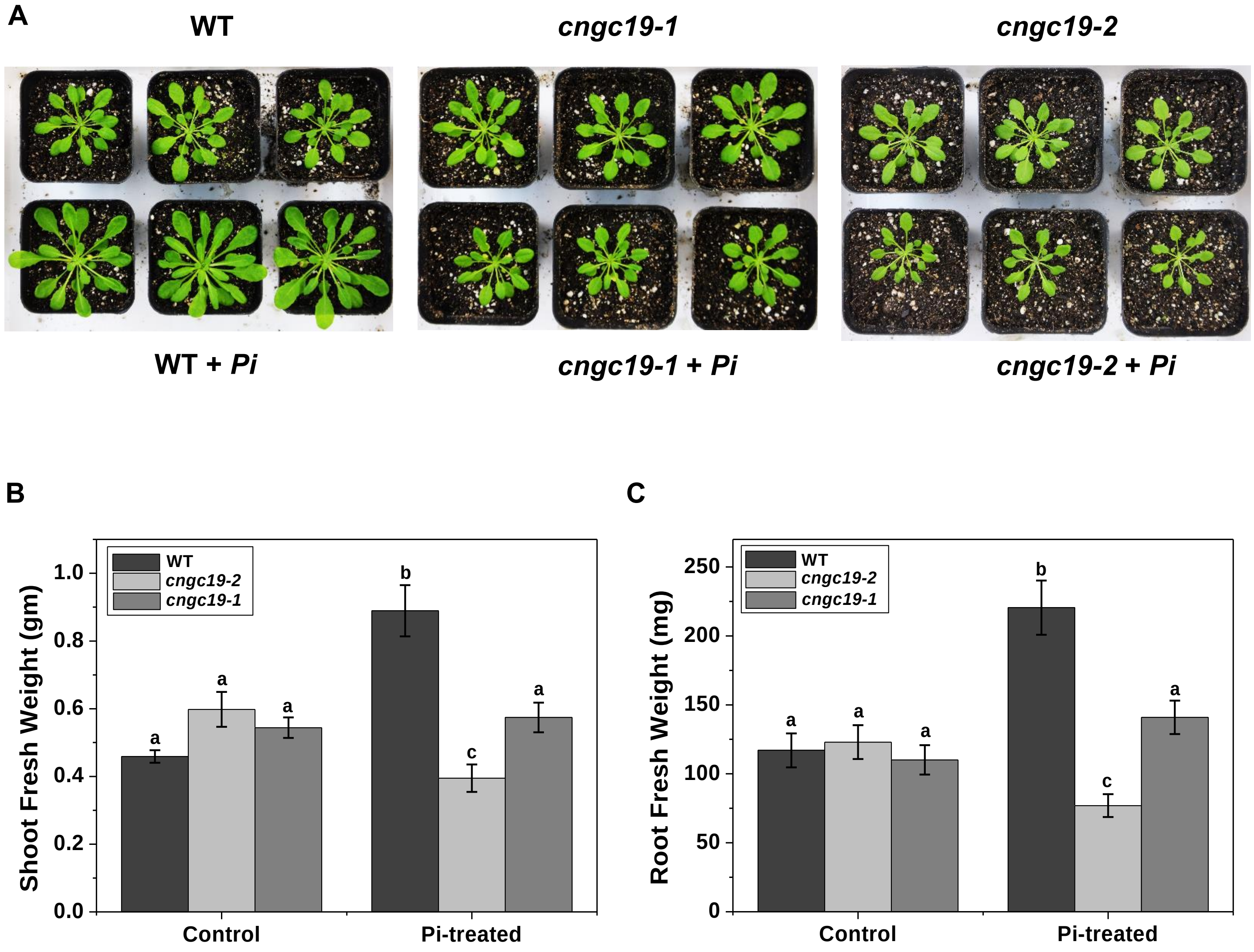

Figure S2. *P. indica* co-cultivation of WT and *cngc19* mutants on soil

A. Plants after 42 dpi co-cultivation with *P. indica* (1% w/w) on soil. Growth phenotype of WT, *cngc19-2* and *cngc19-1* were measured. **B and C.** Shoot and root fresh weight of WT and *cngc19* mutants on soil. Data shown is Mean ± SE (N=24) and the experiment was repeated twice. Different alphabets represent statistically significant difference among WT, *cngc19-2* and *cngc19-1*, calculated by one-way ANOVA with a post hoc Tukey Test (P≤ 0.001).

Figure S3

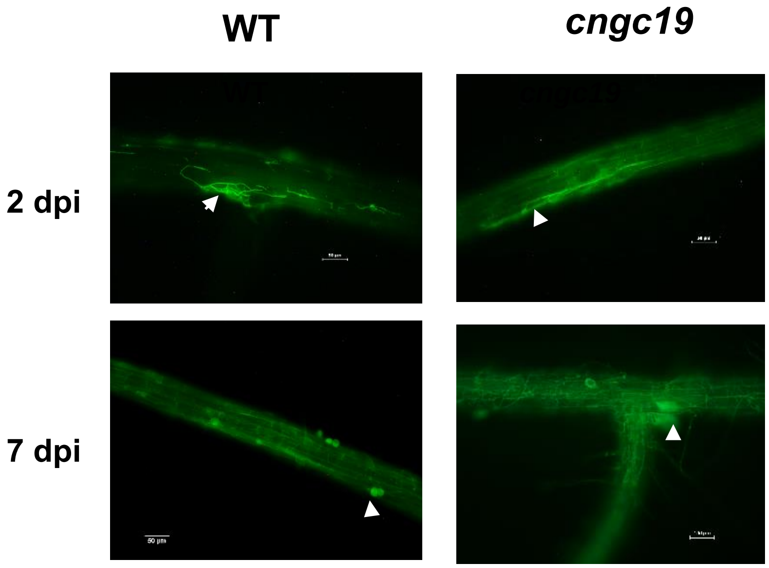

Figure S3. Colonization pattern of *P. indica* on WT and *cngc19*

GFP-tagged *P. indica* was co-cultivated with WT and *cngc19-2.* The roots were harvested for visualizing *P. indica*-colonization at 2 and 7 dpi and observed under fluorescence microscopy.

Figure S4

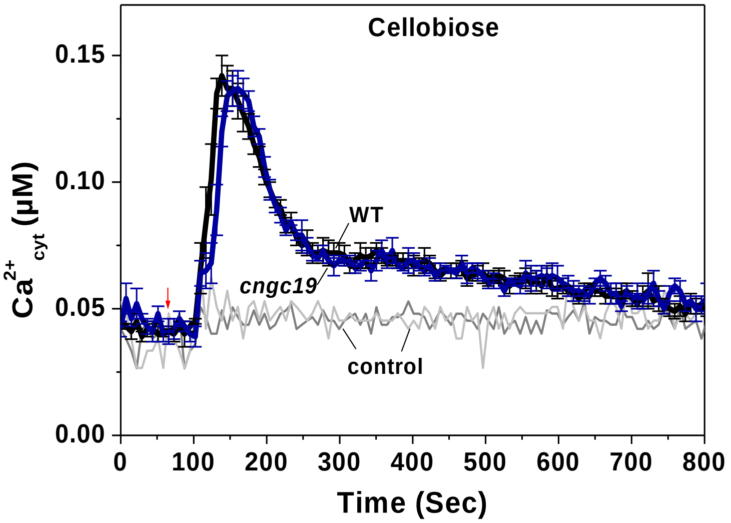

Figure S4. Cytosolic Ca^{2+} levels upon treatment with *P. indica* elicitors

Elevation in cytosolic Ca^{2+} concentration $[Ca^{2+}]_{cyt}$ induced by cellobiose (100 µM) treatment in roots of 10d old WT (Aeq) and *cngc19**aeq line (N=5). Data shown is Mean ± SE. The experiment was repeated thrice with similar results and the figure is representative data from one experiment. Water was used as a control and gave background readings in WT and *cngc19*.

Figure S5

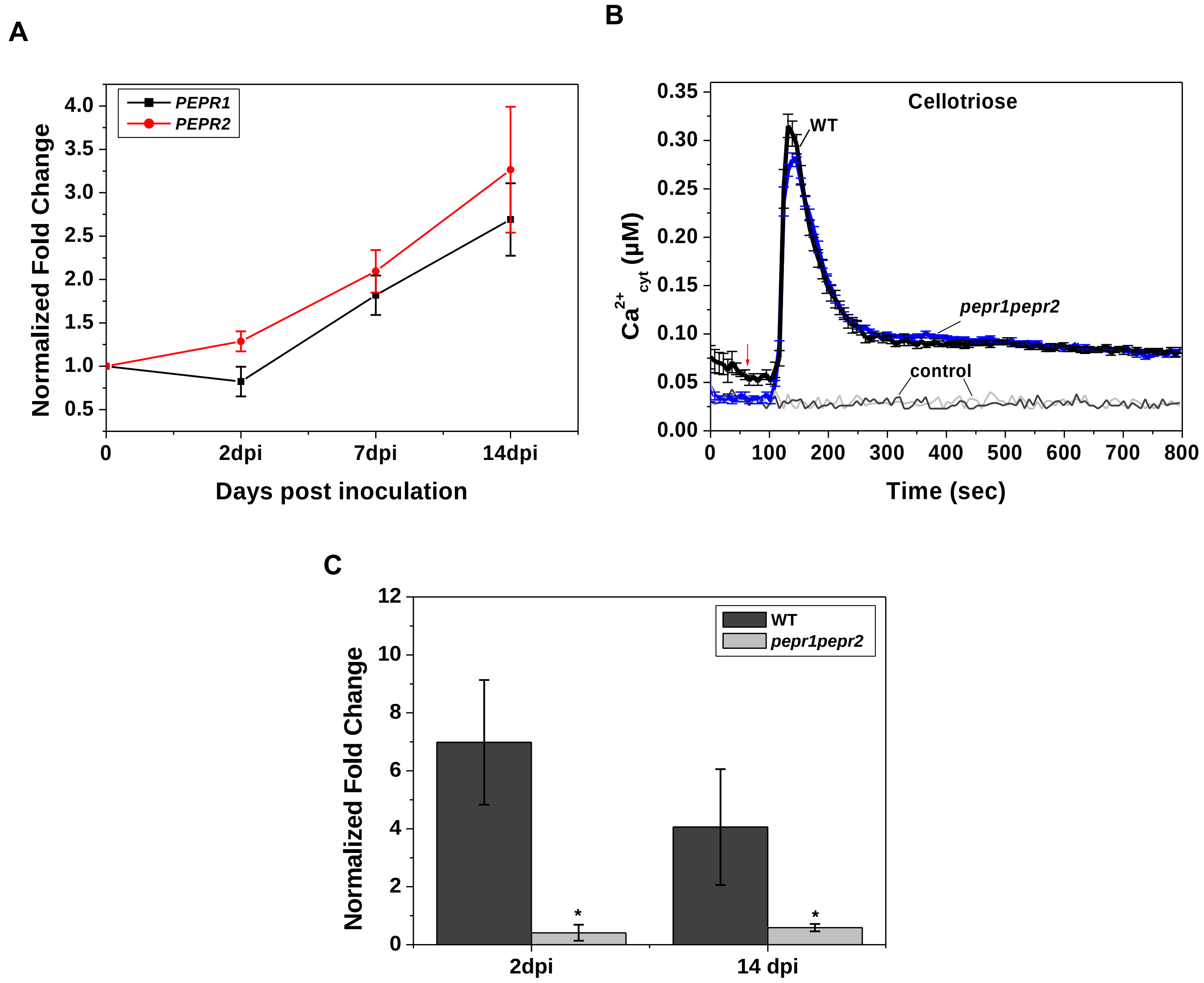

Figure S5. Role of PEPR-signaling in *P. indica* interaction

A. *PEPR1* and *PEPR2* expression during *P. indica*-colonization in WT for 2, 7 and 14dpi. Fold change is the Mean ± SE. In each sample, four seedlings were pooled (N=4). Transcripts level was normalized by *AtActin2* mRNA. **B.** Cytosolic calcium measurement in *pepr1 pepr2* upon treatment of cellotriose. Elevation in cytosolic Ca^{2+} concentration (Ca^{2+}_{cyt}) induced by cellotriose (10 µM) treatment in roots of 10d old WT (Aeq) and *pepr1 pepr2**aeq. Data are shown as the Mean ± SE

(N=5). The experiment was repeated twice with similar results and the figure is representative data from one experiment. Water was used as a control and gave background readings in WT and *pepr1pepr2*. Red arrow indicates time point of elicitor treatment. **C.** *VSP2* expression during *P. indica*-colonization in WT and *pepr1 pepr2* mutants. Fold change is the Mean ± SE. In each sample, four seedlings were pooled (N=4). Transcripts level was normalized by *AtActin2* mRNA. Asterisk (*) indicates a significant difference between co-cultivated WT and *pepr1 pepr2*, calculated by 2 tailed Student's t-test at P≤ 0.05.

Figure S6

A

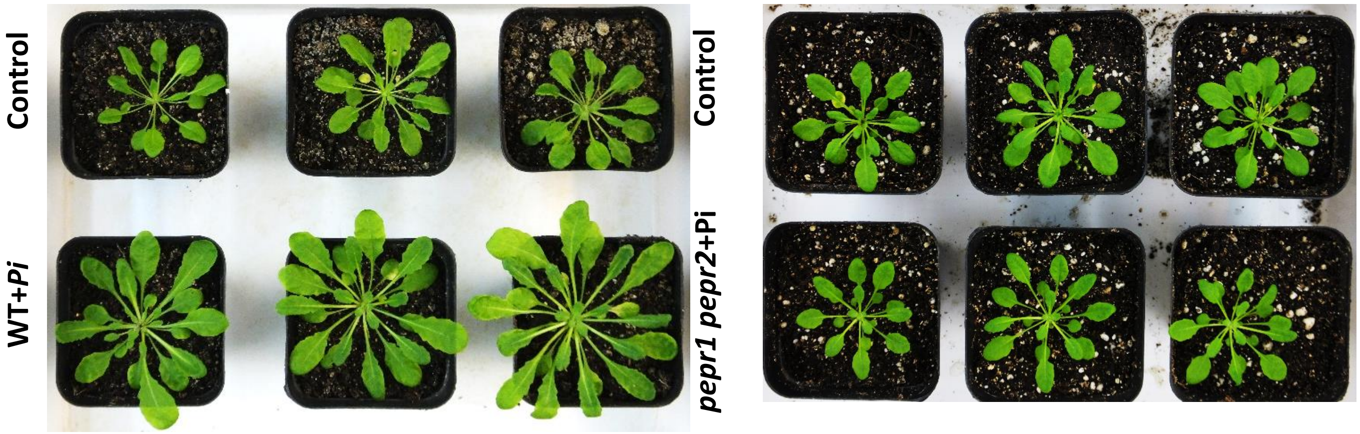

B

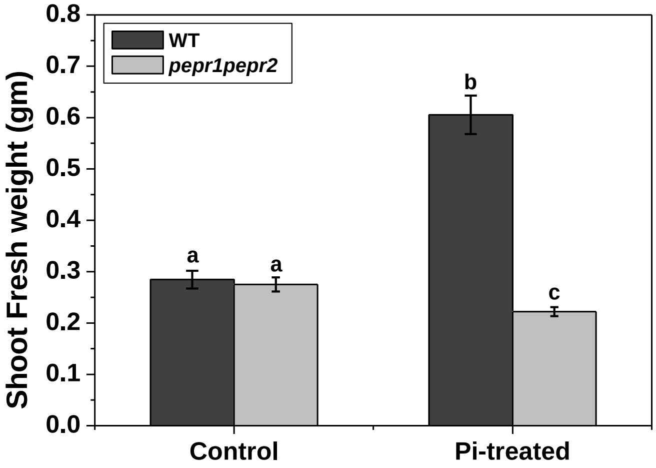

Figure S6. Effect of *P. indica*-colonization on *pepr1 pepr2* double mutant

A. *P. indica* co-cultivation on WT and *pepr1 pepr2* on soil. Representative image of plants after 42 dpi co-cultivation with *P. indica* (1% w/w) on soil. Growth phenotype of WT and *pepr1 pepr2* were measured. **B.** Shoot fresh weight of WT and *pepr1 pepr2* on soil. Data shown is Mean ± SE (N=24). Different alphabets represent statistically significant difference among WT and *pepr1 pepr2*, calculated by one-way ANOVA with a post hoc Tukey Test (P≤ 0.001).

Figure S7

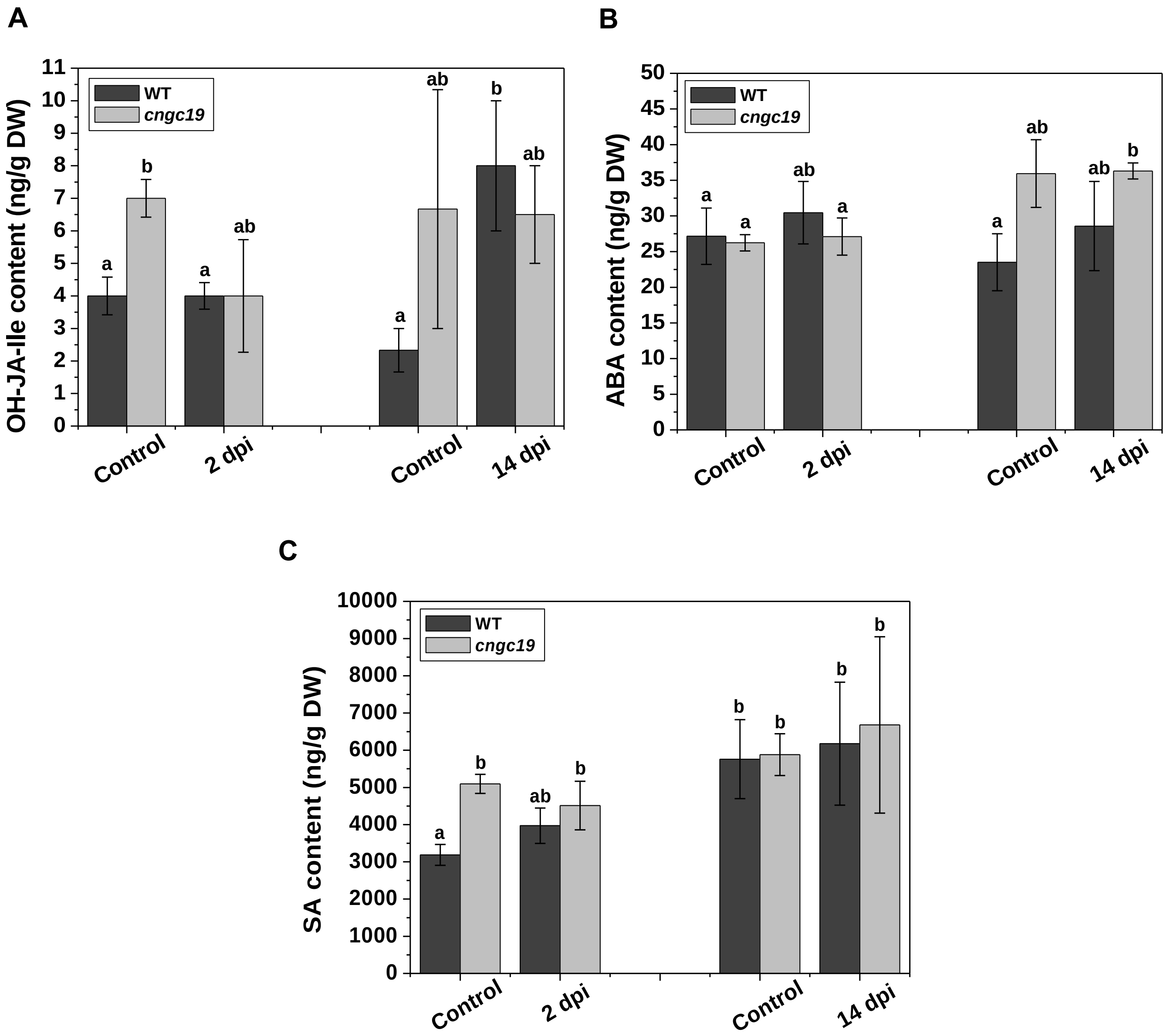

Figure S7. Phytohormone levels in WT and *cngc19-2* during *P. indica* interaction

A. OH-JA-Ile **B.** OH-JA **C.** ABA **D.** SA levels depicted as Mean ± SE. The experiment was performed with 3 biological replicates (N=3). Each replicate is a pool of 40 seedlings. Different alphabets represent statistically significant difference among WT and *cngc19-2*, calculated by one-way ANOVA with a post hoc Tukey Test (P≤ 0.05).

Figure S8

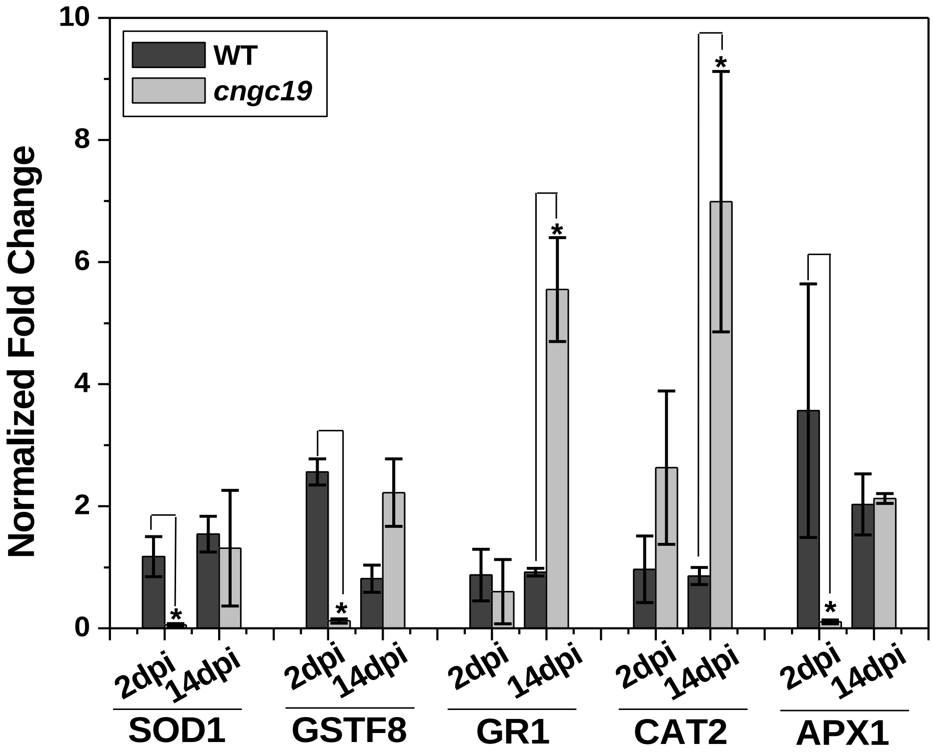

Figure S8: Relative gene expression of additional ROS related genes in WT and *cngc19* seedlings after *P. indica* inoculation: Expression of antioxidant system marker genes in *P. indica* co-cultivated seedlings in Arabidopsis wild-type (black) and *cngc19-2* (grey) plants at early (2 dpi) and late (14 dpi) time points. Fold change was calculated over corresponding non-treated control. Fold change is the Mean ± SE. N=3, with 6 seedling per replicate. Transcripts level was normalized by *AtActin2* mRNA. Asterisk (*) indicates a significant difference between *P. indica* co-cultivated WT and *cngc19*, calculated by 2 tailed Student's *t*-test at $P \leq 0.05$.
